# Effect of hyperthermia on simulated muscle activation in female when crossing obstacle

**DOI:** 10.1038/s41598-024-61536-y

**Published:** 2024-05-09

**Authors:** I.-Lin Wang, Chin-Yi Gu, Tze-Huan Lei, Yu Su, Shun Yao, Toby Mündel, Shiwei Mo

**Affiliations:** 1 Laboratory of Human Kinesiology & Performance, School of Physical Education, Shenzhen University, Guangdong, People’s Republic of China; 2 Graduate Institute, College of Physical Education, Hubei Normal University, Hubei, People’s Republic of China; 3 Beijing Deanwell Technology Co., Ltd, Beijing, People’s Republic of China; 4 Shanghai Hebin Rehabilitation Hospital, Shanghai, People’s Republic of China; 5 Department of Kinesiology, Brock University, St. Catharines, Canada

**Keywords:** Muscle simulation, Hyperthermia, Core temperature, Obstacle height, Gait, Biotechnology, Computational biology and bioinformatics, Neuroscience, Physiology, Anatomy

## Abstract

It is well known that hyperthermia greatly impairs neuromuscular function and dynamic balance. However, whether a greater level of hyperthermia could potentially alter the lower limb simulated muscle activation when crossing an obstacle in female participants remains unknown. Therefore we examined the effect of a systematic increase in oral temperature on lower limb simulated muscle activation when crossing an obstacle in female participants. Eighteen female participants were recruited where they underwent a control trial (Con) and two progressive passive heating trials with Δ 1°C and Δ 2°C increase of oral temperature (T_oral_) using a 45°C water bath. In each trial, we assessed lower limb simulated muscle activation when crossing an obstacle height of 10%, 20%, and 30% of the participant’s leg length and toe-off, toe-above-obstacle and heel-strike events were identified and analyzed. In all events, the lower limb simulated muscle activation were greater in Δ2°C than Δ1°C and Con when both leading and trailing limbs crossed the obstacle height of 20% and 30% leg length (all *p* < 0.001). However, the lower limb simulated muscle activation were not different between Δ1°C and Con across all obstacle heights (*p* > 0.05). This study concluded that a greater level of hyperthermia resulted in a greater lower limb simulated muscle activation to ensure safety and stability when females cross an obstacle height of 20% leg length or higher.

## Introduction

It is well known that a greater level of hyperthermia during exercise exacerbates the development of both central^1^ and peripheral fatigue^2^. The direct consequence of hyperthermia-induced central and peripheral fatigue is the impairment of neuromuscular function due to the reduction of afferent drive to the central nervous system and subsequent reduction to efferent drive from the central nervous system to the skeletal muscle^3^. A typical illustration of neuromuscular function impairment is the reduction of sustained maximal muscle contraction^4^ as well as reducing both static and dynamic balances^5^. This impairment of neuromuscular function greatly increases the risk of falling or directly contributes towards greater musculoskeletal injuries in our daily activities.

Obstacle crossing is quite common and inevitable in daily life, such as overcoming barricades. In comparison to level walking, locomotor control system faces greater challenge in terms of foot clearance and posture stability during obstacle crossing^6^. Particularly, such change increases with the height of the obstacle ^7^. Safely crossing an obstacle requires not only precise motor control of the swing limb clearing the obstacle to avoid tripping or colliding but also stable support of the stance limb^8^. During obstacle crossing, insufficient limb strength would compromise dynamic stability and increase the risk of falling during the single-leg support phase^9^. Therefore, muscular strength is essential to successfully cross an obstacle. Crossing obstacles during walking necessitates a higher level of neuromuscular activation compared to level walking^10^. The increase of oral temperature (T_oral_) could greatly alter the lower limb simulated muscle activation throughout obstacle crossing as previous studies indicated that the static and dynamic balance were greatly impaired with increasing T_oral_^11^. However, whether the increase of T_oral_ affects the lower limb simulated muscle activation during obstacle crossing has not been investigated by previous studies to date. Furthermore, females have poorer dynamic balance than males and are more likely to increase the risk of falling even in thermo-neutral environment^12^. When performing exercise in heat with the rise of oesophageal and rectal temperatures, females have a lower evaporative cooling capacity compared to males^13^ and may therefore have a greater falling risk as the rise of T_oral_ may directly impair both dynamic and static balance ability. Our recent research on the impairment of dynamic balance by elevated oral temperature suggested that under a 2 °C rise of oral temperature, the increased angles of the leading limb joints when crossing higher obstacles resulted in an increase of the toe-clearance to the obstacle because the body responded by elevating the limb in order to cross safely, which was likely to be further supported by a greater activation of the muscles activities of the lower limbs^11^. It is possible that a small elevation of T_oral_ (i.e., 1℃ rise of T T_oral_ or above) could greatly alter the lower limb simulated muscle activation crossing the different obstacle heights and could potentially result in greater falling risk due to the imbalance of agonist and antagonist muscles contraction in the lower limbs. However, previous studies did not investigate the rise of T_oral_ on lower limb simulated muscle activation in female populations during obstacle crossing at various heights and thus warrants further investigation.

Computer simulation of musculoskeletal models has been widely used to analyze and record human movement^14^. The musculoskeletal simulation could simulate and calculate muscle activation that EMG cannot be detected during lower limb activities^15^. It is a useful tool for exploring skeletal muscle activity during walking (i.e., obstacle crossing). Previous studies have used musculoskeletal modeling to understand the impact of various musculoskeletal characteristics on gait and biomechanics during walk^16^. The muscle simulation results from previous studies indicated that the hip and knee extensors provide trunk support in the early stance phase of walking, and the soleus and rectus femoris support trunk propulsion in the late stance phase^17^. The hamstring muscles decelerate the legs in late swing phase and increase the energy absorption of the legs in early stance phase^17^ to maintain a stable gait. However, previous studies only explored the activation of lower limb main muscles during crossing obstacles and neglected the small muscle groups of lower limbs under thermoneutral condition^16^. Therefore, the purpose of this study is to investigate changes in T_oral_ on lower limb simulated muscle activation when crossing obstacles through musculoskeletal simulation. We hypothesized that the increase of T_oral_ by Δ2°C would result in a greater lower limb simulated muscle activation when crossing obstacles of various heights. Resolving those issues from above could help to prevent falling risk for the female population when performing balance related tasks in the heat environment with the rise of T_oral_.

## Materials and methods

### Participants

This study involved eighteen healthy female participants, with an average age of 22.4 ± 2.2 years, height of 166.0 ± 5.5 cm, weight of 54.6 ± 6.7 kg, and leg length of 90.1 ± 4.0 cm. None of the participants had any neurological or musculoskeletal conditions affecting their gait. Informed written consent was obtained from all participants or their legal guardians, confirming their voluntary participation in this research. The study was approved by the Institutional Review Board of Jilin Sports University (Approval No: JLSU-IRB2020002), ensuring adherence to ethical standards in line with the Declaration of Helsinki.

### Experiment design

All participants were required to attend three experimental trials: (1) a control trial without heating (Con); using a 45℃-water bath to increase sublingual temperature (T_oral_) by (2) Δ1℃ and (3) Δ2℃ from baseline to evaluate the effects of hyperthermia on the lower limb simulated muscle activation during different events of obstacle crossing. The three experimental trials were conducted in Jilin Province from autumn to winter when the ambient temperature was below 5°C. Each trial was separated 48 h apart. In order to minimize the influence of circadian rhythm and thermic effect of food on body temperature fluctuations, all experiments were carried out in the morning between 8 and 11 am and performed 2 h postprandial. None of the participants had spent any time in warm weather at least a month prior to the study. Moreover, all participants avoided strenuous exercise, coffee, and alcohol 48 h before each experiment. All experiments were conducted in the early follicular phase to avoid the increase of body temperature and the potential influence on proprioception^18^.

### Protocol

#### Passive heating

Prior to passive heating, euhydration was encouraged by asking participants to consume a premeasured bolus of water (1% of their bodyweight) 2 h prior to the experiment. All participants entered the room with the ambient temperature and passive heating was taken place in the environment of 21°C and 50% relative humidity respectively. After entering the room, participants removed their clothes, put on a swimsuit, and sat on a chair for 10 min to obtain baseline measurements of T_oral_. Throughout the entire trial, T_oral_ (Measurement Computing, Norton, USA) was recorded continuously using data loggers (Supplemental 1). Thereafter, the participants submerged themselves into the bathtub (50 cm diameter *65 cm height) with a water temperature of 45°C and only their head above the water surface^11^. After reaching the specified T_oral_ (Δ1℃ and Δ2℃), participants towel dried themselves. A thermistor was placed inside the oral cavity and participants were not allowed to open their mouth throughout the whole passive heating process. Finally, female researchers accompanied the participants with the obstacle crossing area within one minute to perform obstacle crossing.

#### Crossing obstacle

Participants were allowed to familiarize themselves with the walkway and leg length was measured before passive heating to adjust their starting position and the corrected height of obstacles to ensure the correct limb to cross the obstacle. Leg length was defined as the distance from the ipsilateral anterior superior iliac spine to the medial malleolus^19^. After the passive heating trial, participants entered the mechanics laboratory and walked at a self-selected speed to cross the height-adjustable obstacles on the sidewalk. All participants completed three successful experimental trials. Each trial had three following conditions: (1) crossing an obstacle at a height of 30% of the leg length, (2) crossing an obstacle at a height of 20% of the leg length, (3) crossing an obstacle at a height of 10% of the leg length. All of these conditions were randomised and counter balanced.

### Data collection and analysis

**Figure 1:**
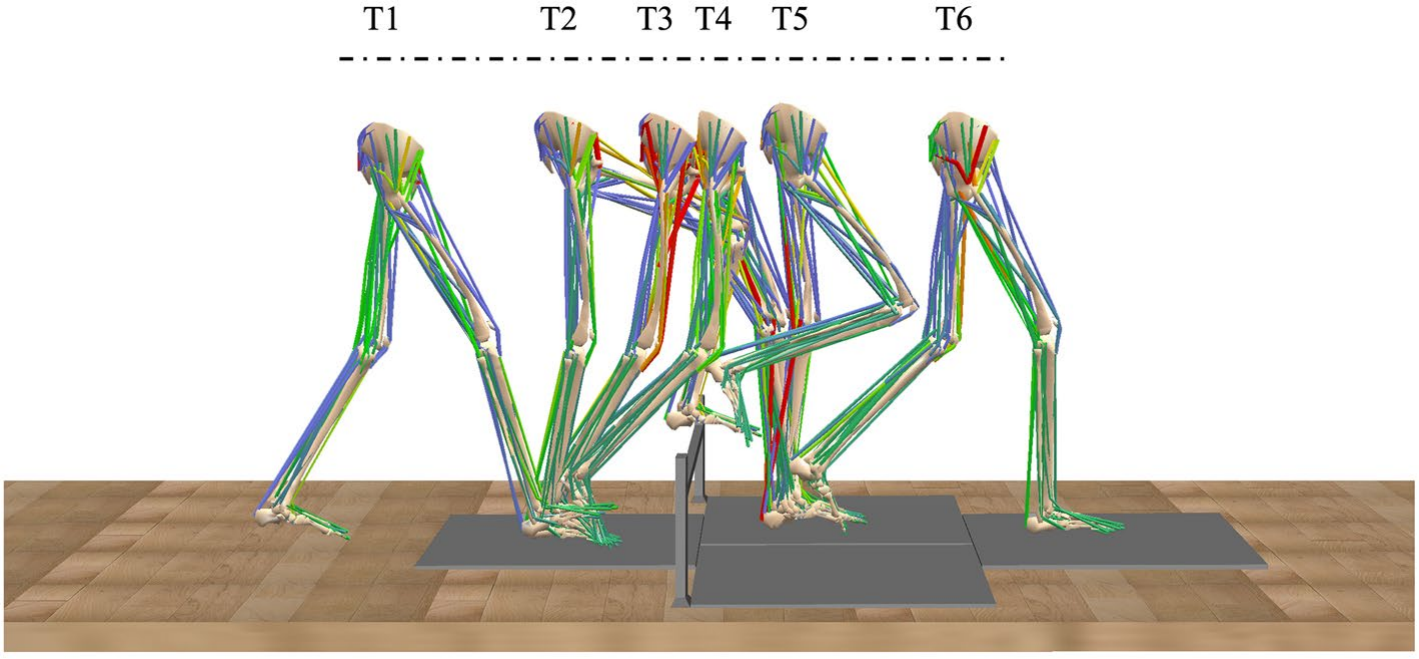
Staging of leading and trailing limbs when crossing an obstacle with height of 10%, 20% and 30% leg length. (T1: Trailing heel-strike; T2: Leading toe-above obstacle; T3: Leading heel-strike; T4: Trailing toe-off; T5: Trailing toe-above obstacle; T6: Leading toe-off).

Two infrared reflective markers were placed on either end of the tube to define the position of the obstacle. A modified Simple Helen Hayes model with 20 reflective markers were secured over selected anatomic landmarks to track the motion of the body segments. A 10-camera system (SMART-DX400, BTS Bioengineering, Milano, Italy) was used to capture the motion with a sampling rate of 100 Hz and a fourth-order Butterworth filter with a cut-off frequency of 5 Hz for low-pass filtering. Four force plates (BTS P6000, BTS Bioengineering, Milano, Italy) were used at a sampling frequency of 200 HZ to collect the ground reaction forces (GRF). The 2nd and 3rd plates were arranged in parallel, subsequently, they were arranged in series with the 1st and 4th plates. GRF of the trailing limb before and after crossing the obstacle were collected with the 1st and 4th force plates, the leading limb after crossing the obstacle were collected with the 2nd or 3rd plates (which one to use depends on which side of the limb is the leading limb)^11,20^. A kinematic model was generated by defining the skeletal segments in the static trial. CusToM toolbox in MATLAB were used to calculate dependent variable^21^, a full body musculoskeletal model, which has been applied to the gait analysis was generated including 17 rigid body segments connected by 14 joints to adapt height and weight of each participant, meanwhile, using the body segment inertia parameters to calibrate segment masses and inertia^11^. The musculoskeletal simulation enables users to calculate inverse kinematics and inverse dynamics using motion capture data. Muscle activation are estimated by determining a distribution that aligns with joint torques and reflects the strategy of the central nervous system^22^. Analysed and calculated lower limb 33 muscles activation in six events (Fig. 1): Trailing heel-strike (T1); Leading toe-above obstacle (T2); Leading heel-strike (T3); Trailing toe-off (T4); Trailing toe-above obstacle (T5); Leading toe-off (T6). The 33 muscles were Pectineus (PEC); Quadratus Femoris (QF); Piriformis (PIRI); Gluteus Minimus (GMIN); Gluteus Medius (GMED); Gluteus Maximus (GMAX); Adductor Brevis (AB); Adductor Longus (AL); Gemellus Superior (GS); Gemellus Inferior (GI); Obturator Externus Muscle (OEM); Obturator Internus Muscle (OIM); Sartorius (SAR); Rectus Femoris (RF); Vastus Intermedius (VI); Vastus Medialis (VM); Vastus Lateralis (VL); Adductor Magnus (AM); Semimembranosus (SM); Semitendinosus (ST); Biceps Femoris short head (BFSH); Biceps Femoris long head (BFLH); Tensor Fasciae Latae (TFL); Gracilis (GRA); Flexor Hallucis Longus (FHL); Flexor Digitorum Longus (FDL); Gastrocnemius (GAS); Soleus (SOL); Tibialis Posterior (TP); Tibialis Anterior (TA); Extensor Hallucis Longus (EHL); Extensor Digitorum Longus (EDL); Peroneus Brevis (PB).

### Statistical analysis

All statistical analyses were performed in MATLAB software (Version 2019a, MathWorks Inc., Natick, MA). All data were analysed by two-way repeated ANOVA (3 T_oral_: Con, Δ1°C, Δ2°C × 3 heights: 10, 20, and 30% of leg length); in the case of where statistical interactions occurred, pairwise comparisons were made using Bonferroni multiple comparisons. Significance level was set at *p* < 0.05. The modified Cohen scale was used to determine the effect size of the three drop height variations, < 0.2 means slight difference, 0.2–0.6 means small difference, 0.6–1.2 means medium difference and > 1.2 means large difference^23^.

## Results

The simulated muscle activation of lower limb were greater in Δ2℃ than in Δ1℃ and Con when crossing obstacles during T1-T6 events in leading or trailing limb (All *p* < 0.05, Tables 1, 2, and 3, Supplemental 2). Specifically, when crossing obstacle heights of 20% and 30% leg length, simulated muscle activation of leading or trailing limbs were greater in Δ2°C than in Δ1℃ and Con except for 10% leg length heights. Furthermore, lower limb simulated muscle activation were not different between Δ1°C and Con at crossing obstacle heights of 10%, 20%, and 30% leg length, respectively (All *p* < 0.05, Tables 1, 2, and 3, Supplemental 2).

**Table 1:** Muscle activation when leading (T6) and trailing (T4) limbs toe-off event at three T_oral_ (Con, Δ1℃, Δ2℃) and heights (10%, 20%,30%).

Characteristic	Treatment	Obstacle height (10%LL)	Obstacle height (20%LL)	Obstacle height (30%LL)	*p-*values		
					Main effects (Height)	Main effects (T_Oral_)	Interaction (T_Oral_ x Height)
Gluteus minimus							
Leading	Con	3.78 ± 0.57	3.80 ± 0.54	3.87 ± 0.54	0.002^**‡**^	0.002^**†**^	0.036*
	Δ1℃	3.83 ± 0.56	3.88 ± 0.53	3.93 ± 0.55			
	Δ2℃	4.06 ± 0.52	4.46 ± 0.55	4.55 ± 0.50			
Trailing	Con	3.84 ± 0.44	4.24 ± 0.53	4.59 ± 0.51	< 0.001^**‡**^	< 0.001^**†**^	0.003*
	Δ1℃	4.04 ± 0.51	4.31 ± 0.65	4.77 ± 0.66			
	Δ2℃	4.33 ± 0.63	5.66 ± 0.77	5.94 ± 0.83			
Gluteus medius							
Leading	Con	6.74 ± 1.33	7.08 ± 1.61	7.11 ± 1.43	0.238	0.387	0.982
	Δ1℃	7.01 ± 1.49	7.14 ± 1.58	7.19 ± 1.55			
	Δ2℃	7.34 ± 1.8	7.60 ± 1.84	7.76 ± 1.34			
Trailing	Con	13.36 ± 3.27	13.85 ± 3.8	15.58 ± 3.33	< 0.001^**‡**^	< 0.001^**†**^	0.002*
	Δ1℃	14.92 ± 3.93	15.91 ± 4.52	17.61 ± 4.91			
	Δ2℃	15.78 ± 4.23	20.42 ± 5.21	23.39 ± 5.46			
Adductor longus							
Leading	Con	1.83 ± 0.74	2.03 ± 0.69	2.15 ± 0.42	0.141	0.33	0.980
	Δ1℃	1.95 ± 0.88	2.08 ± 0.75	2.21 ± 0.32			
	Δ2℃	2.21 ± 1.19	2.28 ± 0.87	2.34 ± 0.54			
Trailing	Con	0.46 ± 0.21	0.51 ± 0.21	0.76 ± 0.37	< 0.001^**‡**^	< 0.001^**†**^	0.049*
	Δ1℃	0.63 ± 0.37	0.71 ± 0.44	0.97 ± 0.47			
	Δ2℃	0.72 ± 0.53	1.29 ± 0.69	1.65 ± 0.79			
Adductor magnus							
Leading	Con	1.52 ± 0.55	1.73 ± 0.74	1.57 ± 0.41	0.053	0.279	0.966
	Δ1℃	1.56 ± 0.57	1.71 ± 0.8	1.51 ± 0.41			
	Δ2℃	1.83 ± 0.75	1.95 ± 0.7	1.78 ± 0.62			
Trailing	Con	1.300 ± 0.57	1.40 ± 0.69	1.50 ± 0.61	0.168	0.278	0.998
	Δ1℃	1.43 ± 0.61	1.51 ± 0.74	1.69 ± 0.72			
	Δ2℃	1.49 ± 0.82	1.65 ± 0.78	1.77 ± 0.76			
Quadratus femoris							
Leading	Con	2.15 ± 0.48	2.06 ± 0.41	2.35 ± 0.38	< 0.001^**‡**^	< 0.001^**†**^	0.033*
	Δ1℃	2.14 ± 0.42	2.15 ± 0.35	2.39 ± 0.34			
	Δ2℃	2.38 ± 0.32	2.62 ± 0.38	2.90 ± 0.35			
Trailing	Con	1.38 ± 0.36	1.47 ± 0.47	1.65 ± 0.41	< 0.001^**‡**^	< 0.001^**†**^	0.015*
	Δ1℃	1.57 ± 0.44	1.53 ± 0.55	1.83 ± 0.54			
	Δ2℃	1.60 ± 0.58	2.18 ± 0.61	2.55 ± 0.71			
Adductor brevis							
Leading	Con	1.11 ± 0.51	1.16 ± 0.48	1.20 ± 0.47	0.13	0.405	0.965
	Δ1℃	1.15 ± 0.50	1.20 ± 0.62	1.28 ± 0.69			
	Δ2℃	1.30 ± 0.61	1.38 ± 0.72	1.48 ± 0.67			
Trailing	Con	0.35 ± 0.20	0.43 ± 0.33	0.54 ± 0.37	< 0.001^**‡**^	< 0.001^**†**^	0.026*
	Δ1℃	0.44 ± 0.36	0.51 ± 0.38	0.61 ± 0.36			
	Δ2℃	0.57 ± 0.41	0.99 ± 0.63	1.42 ± 0.72			
Obturator internus muscle							
Leading	Con	3.15 ± 0.25	3.16 ± 0.35	3.26 ± 0.32	< 0.001^**‡**^	< 0.001^**†**^	0.048*
	Δ1℃	3.19 ± 0.3	3.2 ± 0.33	3.3 ± 0.36			
	Δ2℃	3.39 ± 0.32	3.64 ± 0.29	3.78 ± 0.36			
Trailing	Con	2.53 ± 0.49	2.74 ± 0.55	3.21 ± 0.57	< 0.001^**‡**^	< 0.001^**†**^	0.048*
	Δ1℃	2.73 ± 0.54	2.95 ± 0.66	3.32 ± 0.61			
	Δ2℃	2.80 ± 0.62	3.55 ± 0.71	4.13 ± 0.71			
Obturator externus muscle							
Leading	Con	2.99 ± 0.49	3.37 ± 0.54	4.05 ± 0.59	< 0.001^**‡**^	< 0.001^**†**^	0.004*
	Δ1℃	3.05 ± 0.67	3.42 ± 0.58	4.11 ± 0.59			
	Δ2℃	3.5 ± 0.77	4.22 ± 0.57	5.37 ± 0.59			
Trailing	Con	2.28 ± 0.58	2.20 ± 0.56	3.14 ± 0.57	< 0.001^**‡**^	< 0.001^**†**^	0.049*
	Δ1℃	2.53 ± 0.61	2.71 ± 0.78	3.33 ± 0.73			
	Δ2℃	2.86 ± 0.71	3.53 ± 0.87	4.49 ± 0.88			
Pectineus							
Leading	Con	3.18 ± 0.69	3.17 ± 0.78	3.64 ± 0.51	< 0.001^**‡**^	< 0.001^**†**^	0.007*
	Δ1℃	3.21 ± 0.79	3.23 ± 0.82	3.71 ± 0.49			
	Δ2℃	3.83 ± 0.75	4.67 ± 0.70	5.24 ± 0.70			
Trailing	Con	1.19 ± 0.47	1.33 ± 0.54	2.08 ± 0.51	< 0.001^**‡**^	< 0.001^**†**^	0.037*
	Δ1℃	1.22 ± 0.67	1.51 ± 0.65	2.22 ± 0.82			
	Δ2℃	1.43 ± 0.78	2.4 ± 0.93	3.24 ± 0.93			
Gemellus inferior							
Leading	Con	0.69 ± 0.08	0.72 ± 0.11	0.8 ± 0.09	< 0.001^**‡**^	< 0.001^**†**^	0.018*
	Δ1℃	0.69 ± 0.09	0.73 ± 0.11	0.82 ± 0.10			
	Δ2℃	0.75 ± 0.12	0.84 ± 0.09	0.95 ± 0.09			
Trailing	Con	0.47 ± 0.08	0.5 ± 0.09	0.6 ± 0.09	< 0.001^**‡**^	< 0.001^**†**^	0.049*
	Δ1℃	0.52 ± 0.09	0.55 ± 0.10	0.63 ± 0.12			
	Δ2℃	0.53 ± 0.09	0.66 ± 0.11	0.75 ± 0.13			
Gemellus superior							
Leading	Con	0.63 ± 0.07	0.67 ± 0.09	0.71 ± 0.07	< 0.001^**‡**^	0.001^**†**^	0.022*
	Δ1℃	0.63 ± 0.08	0.68 ± 0.10	0.74 ± 0.09			
	Δ2℃	0.68 ± 0.13	0.77 ± 0.10	0.86 ± 0.09			
Trailing	Con	0.35 ± 0.05	0.41 ± 0.07	0.42 ± 0.07	< 0.001^**‡**^	< 0.001^**†**^	0.048*
	Δ1℃	0.40 ± 0.06	0.43 ± 0.08	0.44 ± 0.09			
	Δ2℃	0.42 ± 0.10	0.53 ± 0.09	0.58 ± 0.09			
Gluteus maximus							
Leading	Con	5.01 ± 0.37	4.61 ± 0.26	4.14 ± 0.30	< 0.001^**‡**^	< 0.001^**†**^	0.003*
	Δ1℃	5.01 ± 0.37	4.66 ± 0.22	4.23 ± 0.28			
	Δ2℃	5.27 ± 0.31	5.13 ± 0.21	4.97 ± 0.18			
Trailing	Con	4.28 ± 0.42	3.46 ± 0.46	2.82 ± 0.48	< 0.001^**‡**^	< 0.001^**†**^	0.047*
	Δ1℃	4.39 ± 0.59	3.72 ± 0.58	3.11 ± 0.56			
	Δ2℃	4.62 ± 0.54	4.31 ± 0.60	3.84 ± 0.64			
Piriformis							
Leading	Con	3.96 ± 0.41	4.12 ± 0.46	4.9 ± 0.46	< 0.001^**‡**^	< 0.001^**†**^	< 0.001*
	Δ1℃	3.99 ± 0.4	4.19 ± 0.4	4.95 ± 0.49			
	Δ2℃	4.19 ± 0.56	5.03 ± 0.46	5.98 ± 0.77			
Trailing	Con	2.33 ± 0.23	2.38 ± 0.29	2.59 ± 0.38	< 0.001^**‡**^	< 0.001^**†**^	< 0.001*
	Δ1℃	2.35 ± 0.26	2.40 ± 0.30	2.64 ± 0.39			
	Δ2℃	2.45 ± 0.23	3.19 ± 0.47	3.43 ± 0.55			
Vastus lateralis							
Leading	Con	0.92 ± 0.43	0.82 ± 0.50	0.77 ± 0.65	0.199	0.589	0.995
	Δ1℃	0.93 ± 0.48	0.86 ± 0.64	0.75 ± 0.67			
	Δ2℃	1.06 ± 0.39	0.96 ± 0.72	0.84 ± 0.64			
Trailing	Con	7.41 ± 1.16	7.53 ± 1.19	7.84 ± 1.21	0.164	0.677	0.994
	Δ1℃	7.49 ± 1.12	7.73 ± 1.17	7.91 ± 1.23			
	Δ2℃	7.55 ± 1.62	7.93 ± 1.38	8.04 ± 1.48			
Vastus medialis							
Leading	Con	0.61 ± 0.23	0.51 ± 0.42	0.51 ± 0.48	0.325	0.509	0.999
	Δ1℃	0.61 ± 0.27	0.55 ± 0.51	0.52 ± 0.49			
	Δ2℃	0.73 ± 0.29	0.63 ± 0.57	0.61 ± 0.57			
Trailing	Con	5.86 ± 1.11	6.04 ± 1.21	6.29 ± 1.18	0.098	0.2	0.980
	Δ1℃	5.91 ± 1.27	6.29 ± 1.34	6.61 ± 1.33			
	Δ2℃	6.39 ± 1.48	6.49 ± 1.49	6.86 ± 1.53			
Vastus intermedius							
Leading	Con	0.59 ± 0.38	0.53 ± 0.41	0.51 ± 0.57	0.331	0.842	0.996
	Δ1℃	0.62 ± 0.42	0.53 ± 0.49	0.51 ± 0.61			
	Δ2℃	0.69 ± 0.39	0.60 ± 0.55	0.54 ± 0.66			
Trailing	Con	5.93 ± 1.33	6.20 ± 1.08	6.50 ± 1.02	0.127	0.24	0.991
	Δ1℃	6.18 ± 1.46	6.51 ± 1.17	6.71 ± 1.14			
	Δ2℃	6.51 ± 1.73	6.75 ± 1.25	6.82 ± 1.22			
Rectus femoris							
Leading	Con	7.85 ± 1.39	7.75 ± 1.78	7.30 ± 1.75	0.062	0.284	0.909
	Δ1℃	7.93 ± 1.65	7.91 ± 1.79	7.55 ± 1.80			
	Δ2℃	8.79 ± 2.06	8.35 ± 2.01	7.99 ± 1.67			
Trailing	Con	11.75 ± 1.74	11.57 ± 1.94	11.34 ± 1.96	0.411	0.093	0.990
	Δ1℃	12.48 ± 2.05	12.36 ± 2.22	12.25 ± 2.32			
	Δ2℃	13.13 ± 2.54	12.97 ± 2.52	12.57 ± 2.59			
Semitendinosus							
Leading	Con	4.70 ± 0.42	4.87 ± 0.53	5.39 ± 0.59	< 0.001^**‡**^	< 0.001^**†**^	0.002*
	Δ1℃	4.76 ± 0.5	4.95 ± 0.54	5.50 ± 0.56			
	Δ2℃	4.93 ± 0.43	5.61 ± 0.48	6.42 ± 0.48			
Trailing	Con	7.11 ± 1.28	7.34 ± 1.34	7.52 ± 1.45	0.069	0.204	0.992
	Δ1℃	7.31 ± 1.58	7.54 ± 1.56	7.79 ± 1.56			
	Δ2℃	7.74 ± 1.76	8.18 ± 1.92	8.36 ± 1.82			
Semimembranosus							
Leading	Con	10.32 ± 1.32	10.24 ± 1.68	10.15 ± 1.54	0.14	0.952	0.86
	Δ1℃	10.46 ± 1.52	10.4 ± 1.73	10.23 ± 1.66			
	Δ2℃	10.4 ± 1.59	10.42 ± 1.65	10.34 ± 1.77			
Trailing	Con	12.48 ± 1.46	12.75 ± 1.78	13.05 ± 1.61	0.295	0.073	0.988
	Δ1℃	13.34 ± 1.98	13.67 ± 2.08	13.73 ± 1.97			
	Δ2℃	13.91 ± 2.06	14.06 ± 2.13	14.16 ± 2.57			
Biceps Femoris long head							
Leading	Con	7.35 ± 0.56	7.46 ± 0.49	8.05 ± 0.63	< 0.001^**‡**^	< 0.001^**†**^	0.034*
	Δ1℃	7.40 ± 0.61	7.48 ± 0.48	8.10 ± 0.64			
	Δ2℃	7.60 ± 0.71	8.19 ± 0.49	8.91 ± 0.56			
Trailing	Con	6.24 ± 0.92	6.06 ± 0.89	5.76 ± 0.73	0.056	0.102	0.987
	Δ1℃	6.49 ± 0.92	6.36 ± 1.06	6.09 ± 1.20			
	Δ2℃	6.74 ± 1.10	6.69 ± 1.27	6.43 ± 1.38			
Biceps femoris short head							
Leading	Con	2.24 ± 0.49	2.61 ± 0.34	5.04 ± 0.56	< 0.001^**‡**^	< 0.001^**†**^	< 0.001*
	Δ1℃	2.26 ± 0.55	2.67 ± 0.32	5.1 ± 0.56			
	Δ2℃	2.46 ± 0.55	3.64 ± 0.38	7.07 ± 0.59			
Trailing	Con	5.46 ± 1.23	5.77 ± 1.46	5.99 ± 1.42	0.212	0.562	0.995
	Δ1℃	5.62 ± 1.33	6.04 ± 1.79	6.17 ± 1.60			
	Δ2℃	5.83 ± 1.57	6.08 ± 1.98	6.36 ± 1.89			
Sartorius							
Leading	Con	3.01 ± 0.46	3.2 ± 0.34	3.62 ± 0.24	< 0.001^**‡**^	< 0.001^**†**^	< 0.001*
	Δ1℃	3.01 ± 0.46	3.23 ± 0.35	3.66 ± 0.26			
	Δ2℃	3.24 ± 0.47	4.29 ± 0.31	5.48 ± 0.32			
Trailing	Con	2.67 ± 0.52	3.10 ± 0.66	3.87 ± 0.65	< 0.001^**‡**^	< 0.001^**†**^	0.035*
	Δ1℃	3.08 ± 0.55	3.33 ± 0.72	4.19 ± 0.73			
	Δ2℃	3.21 ± 0.62	4.41 ± 0.81	5.00 ± 0.82			
Gracilis muscle							
Leading	Con	0.78 ± 0.12	0.82 ± 0.14	1.01 ± 0.21	< 0.001^**‡**^	< 0.001^**†**^	0.036*
	Δ1℃	0.79 ± 0.12	0.84 ± 0.12	1.05 ± 0.17			
	Δ2℃	1.04 ± 0.16	1.14 ± 0.19	1.15 ± 0.16			
Trailing	Con	0.49 ± 0.08	0.60 ± 0.09	0.75 ± 0.11	< 0.001^**‡**^	< 0.001^**†**^	0.02*
	Δ1℃	0.55 ± 0.11	0.62 ± 0.11	0.78 ± 0.12			
	Δ2℃	0.58 ± 0.12	0.75 ± 0.12	0.95 ± 0.13			
Tensor fasciae lata							
Leading	Con	3.70 ± 1.08	3.37 ± 1.62	3.91 ± 1.34	0.051	0.273	0.797
	Δ1℃	3.92 ± 1.81	4.17 ± 1.45	4.70 ± 1.70			
	Δ2℃	4.20 ± 2.31	4.40 ± 2.29	5.07 ± 2.03			
Trailing	Con	7.34 ± 1.21	7.69 ± 1.47	8.12 ± 1.4	0.051	0.457	0.999
	Δ1℃	7.57 ± 1.49	7.87 ± 1.61	8.26 ± 1.90			
	Δ2℃	7.92 ± 1.79	8.21 ± 1.96	8.51 ± 1.91			
Soleus							
Leading	Con	0.01 ± 0.02	0.02 ± 0.04	0.02 ± 0.04	0.266	0.109	0.496
	Δ1℃	0.03 ± 0.06	0.02 ± 0.05	0.02 ± 0.04			
	Δ2℃	0.06 ± 0.10	0.04 ± 0.05	0.02 ± 0.04			
Trailing	Con	4.15 ± 1.22	4.33 ± 1.41	4.26 ± 1.47	0.678	0.549	0.996
	Δ1℃	4.33 ± 1.48	4.51 ± 1.56	4.63 ± 1.66			
	Δ2℃	4.52 ± 1.67	4.64 ± 1.84	4.82 ± 1.95			
Gastrocnemius							
Leading	Con	0.91 ± 0.52	0.90 ± 0.39	1.03 ± 0.64	0.448	0.281	0.998
	Δ1℃	0.91 ± 0.65	0.93 ± 0.69	1.05 ± 0.55			
	Δ2℃	1.11 ± 0.61	1.17 ± 0.84	1.22 ± 0.63			
Trailing	Con	13.01 ± 1.94	13.11 ± 1.88	13.56 ± 1.84	0.226	0.145	0.995
	Δ1℃	13.39 ± 2.00	13.88 ± 2.17	14.08 ± 2.14			
	Δ2℃	13.71 ± 2.12	14.10 ± 2.27	14.49 ± 2.60			
Flexor digitorum longus							
Leading	Con	0.16 ± 0.3	0.17 ± 0.33	0.17 ± 0.27	0.714	0.295	0.838
	Δ1℃	0.2 ± 0.37	0.19 ± 0.38	0.22 ± 0.4			
	Δ2℃	0.44 ± 0.74	0.29 ± 0.54	0.33 ± 0.54			
Trailing	Con	3.13 ± 1.24	3.29 ± 1.32	3.45 ± 2.17	0.517	0.757	0.942
	Δ1℃	3.21 ± 1.86	3.34 ± 1.47	3.54 ± 1.83			
	Δ2℃	3.42 ± 1.53	3.49 ± 1.58	3.76 ± 1.61			
Flexor hallucis longus							
Leading	Con	0.01 ± 0.02	0.01 ± 0.02	0.01 ± 0.03	0.757	0.643	0.938
	Δ1℃	0.01 ± 0.01	0.01 ± 0.03	0.01 ± 0.03			
	Δ2℃	0.02 ± 0.02	0.02 ± 0.02	0.01 ± 0.02			
Trailing	Con	0.31 ± 0.13	0.33 ± 0.12	0.34 ± 0.12	0.127	0.052	0.99
	Δ1℃	0.34 ± 0.15	0.37 ± 0.14	0.39 ± 0.14			
	Δ2℃	0.38 ± 0.15	0.41 ± 0.16	0.44 ± 0.17			
Tibialis posterior							
Leading	Con	0.62 ± 1.36	0.61 ± 1.37	0.31 ± 0.73	0.684	0.785	0.844
	Δ1℃	0.76 ± 1.51	0.91 ± 2.02	0.66 ± 1.41			
	Δ2℃	0.82 ± 1.55	0.58 ± 1.07	0.75 ± 1.70			
Trailing	Con	7.85 ± 1.57	8.14 ± 1.77	8.37 ± 1.61	0.619	0.102	0.996
	Δ1℃	8.31 ± 1.66	8.37 ± 1.79	8.50 ± 1.82			
	Δ2℃	8.74 ± 1.91	8.77 ± 2.39	9.11 ± 2.29			
Peroneus brevis							
Leading	Con	12.55 ± 11.81	11.42 ± 10.49	10.6 ± 10.94	0.919	0.948	0.613
	Δ1℃	12.92 ± 11.61	12.38 ± 10.21	12.24 ± 13.06			
	Δ2℃	11.33 ± 10.45	11.82 ± 10.75	14.33 ± 15.99			
Trailing	Con	18.26 ± 5.40	19.33 ± 5.37	20.70 ± 5.25	0.059	0.205	0.997
	Δ1℃	18.65 ± 6.02	20.03 ± 5.88	21.75 ± 5.73			
	Δ2℃	20.85 ± 6.77	21.38 ± 7.59	23.27 ± 7.58			
Tibialis anterior							
Leading	Con	1.29 ± 0.59	1.16 ± 0.79	1.41 ± 0.77	0.341	0.064	0.972
	Δ1℃	1.45 ± 0.66	1.37 ± 0.78	1.51 ± 0.59			
	Δ2℃	1.78 ± 0.62	1.76 ± 1.09	1.84 ± 0.97			
Trailing	Con	2.39 ± 0.39	2.47 ± 0.65	2.63 ± 0.7	0.294	0.061	0.999
	Δ1℃	2.59 ± 0.56	2.61 ± 0.66	2.76 ± 0.86			
	Δ2℃	2.78 ± 0.69	2.82 ± 0.89	2.98 ± 0.92			
Extensor digitorum longus							
Leading	Con	2.02 ± 0.83	2.15 ± 1.10	2.30 ± 0.93	0.064	0.865	0.931
	Δ1℃	1.94 ± 1.55	2.14 ± 0.72	2.25 ± 0.76			
	Δ2℃	1.95 ± 0.93	2.26 ± 1.19	2.52 ± 0.73			
Trailing	Con	6.77 ± 1.45	6.93 ± 1.35	7.21 ± 1.78	0.383	0.222	0.998
	Δ1℃	7.18 ± 1.59	7.22 ± 1.88	7.59 ± 2.09			
	Δ2℃	7.44 ± 1.75	7.53 ± 2.01	7.96 ± 2.51			
Extensor hallucis longus							
Leading	Con	1.83 ± 0.74	2.02 ± 0.68	2.14 ± 0.41	0.141	0.330	0.980
	Δ1℃	1.95 ± 0.86	2.07 ± 0.74	2.21 ± 0.32			
	Δ2℃	2.21 ± 1.19	2.28 ± 0.87	2.34 ± 0.54			
Trailing	Con	0.7 ± 0.15	0.72 ± 0.17	0.75 ± 0.19	0.249	0.589	0.976
	Δ1℃	0.71 ± 0.2	0.74 ± 0.18	0.77 ± 0.24			
	Δ2℃	0.76 ± 0.28	0.79 ± 0.23	0.81 ± 0.35			

“‡” Main effect of height (*p* < 0.05).

“†” Main effect of T_Oral_ (*p* < 0.05).

“*” Significant T_Oral_ x height interaction effects (*p* < 0.05).

**Table 2:** Muscle activation when leading (T2) and trailing (T5) limbs toe-above the obstacle event a three T_oral_ (Con, Δ1℃, Δ2℃) and heights (10%, 20%,30%).

Characteristic	Treatment	Obstacle height (10%LL)	Obstacle height (20%LL)	Obstacle height (30%LL)	*p-*values		
					Main effects (Height)	Main effects (T_Oral_)	Interaction (T_Oral_ × Height)
Gluteus minimus							
Leading	Con	2.77 ± 0.69	3.01 ± 0.84	2.70 ± 0.66	0.383	0.078	0.779
	Δ1℃	3.01 ± 0.47	3.06 ± 0.75	2.86 ± 0.61			
	Δ2℃	3.22 ± 0.93	3.24 ± 0.65	3.27 ± 0.85			
Trailing	Con	2.84 ± 0.76	2.94 ± 0.70	3.03 ± 0.67	0.065	0.626	0.985
	Δ1℃	2.94 ± 0.49	3.01 ± 0.73	3.12 ± 0.73			
	Δ2℃	2.97 ± 0.69	3.12 ± 0.73	3.27 ± 0.53			
Gluteus Medius							
Leading	Con	2.07 ± 0.59	1.99 ± 0.52	1.92 ± 0.60	0.248	0.059	0.993
	Δ1℃	2.24 ± 0.59	2.06 ± 0.52	2.02 ± 0.68			
	Δ2℃	2.39 ± 0.58	2.31 ± 0.52	2.24 ± 0.76			
Trailing	Con	3.11 ± 0.75	3.09 ± 0.92	2.97 ± 0.77	0.403	0.253	0.998
	Δ1℃	3.31 ± 0.91	3.21 ± 0.91	3.07 ± 0.84			
	Δ2℃	3.51 ± 0.91	3.43 ± 0.86	3.35 ± 0.92			
Adductor longus							
Leading	Con	1.11 ± 1.13	1.10 ± 0.56	1.12 ± 0.74	0.912	0.715	0.994
	Δ1℃	1.11 ± 0.62	1.17 ± 0.88	1.13 ± 0.67			
	Δ2℃	1.22 ± 0.81	1.26 ± 0.91	1.33 ± 0.88			
Trailing	Con	0.88 ± 0.4	0.92 ± 0.77	0.93 ± 0.57	0.834	0.644	1.000
	Δ1℃	0.91 ± 0.61	0.95 ± 0.81	0.97 ± 0.71			
	Δ2℃	1.00 ± 0.66	1.03 ± 0.79	1.11 ± 0.78			
Adductor magnus							
Leading	Con	2.08 ± 0.28	2.11 ± 0.37	2.41 ± 0.35	< 0.001^**‡**^	< 0.001^**†**^	0.043*
	Δ1℃	2.12 ± 0.29	2.27 ± 0.28	2.44 ± 0.36			
	Δ2℃	2.90 ± 0.25	3.03 ± 0.33	3.55 ± 0.36			
Trailing	Con	0.97 ± 0.34	1.04 ± 0.19	1.22 ± 0.20	< 0.001^**‡**^	< 0.001^**†**^	0.046*
	Δ1℃	1.05 ± 0.36	1.05 ± 0.21	1.27 ± 0.25			
	Δ2℃	1.18 ± 0.22	1.45 ± 0.31	1.75 ± 0.39			
Quadratus femoris							
Leading	Con	3.60 ± 0.82	3.59 ± 0.81	3.68 ± 0.71	0.07	0.407	0.527
	Δ1℃	3.63 ± 0.72	3.65 ± 0.90	3.74 ± 0.79			
	Δ2℃	3.73 ± 0.76	3.94 ± 0.86	4.11 ± 0.77			
Trailing	Con	1.79 ± 0.40	1.84 ± 0.28	2.15 ± 0.38	< 0.001^**‡**^	< 0.001^**†**^	< 0.001*
	Δ1℃	1.91 ± 0.41	1.93 ± 0.32	2.18 ± 0.38			
	Δ2℃	2.07 ± 0.48	2.49 ± 0.36	3.08 ± 0.40			
Adductor brevis							
Leading	Con	1.56 ± 0.26	1.49 ± 0.29	1.68 ± 0.26	< 0.001^**‡**^	< 0.001^**†**^	< 0.001*
	Δ1℃	1.57 ± 0.33	1.54 ± 0.32	1.72 ± 0.28			
	Δ2℃	1.77 ± 0.41	2.19 ± 0.29	2.55 ± 0.30			
Trailing	Con	0.53 ± 0.23	0.61 ± 0.12	0.72 ± 0.14	< 0.001^**‡**^	< 0.001^**†**^	0.035*
	Δ1℃	0.55 ± 0.24	0.64 ± 0.15	0.74 ± 0.14			
	Δ2℃	0.67 ± 0.24	0.92 ± 0.15	1.1 ± 0.14			
Obturator Internus Muscle							
Leading	Con	2.84 ± 0.81	2.95 ± 0.72	3.04 ± 0.77	0.592	0.191	0.961
	Δ1℃	3.22 ± 0.61	3.23 ± 0.64	3.24 ± 0.69			
	Δ2℃	3.27 ± 0.74	3.28 ± 0.80	3.35 ± 0.76			
Trailing	Con	2.76 ± 0.61	2.82 ± 0.62	2.91 ± 0.73	0.288	0.328	0.998
	Δ1℃	2.91 ± 0.46	2.93 ± 0.56	3.06 ± 0.76			
	Δ2℃	3.06 ± 0.62	3.10 ± 0.88	3.17 ± 0.68			
Obturator Externus Muscle							
Leading	Con	3.99 ± 0.59	5.04 ± 0.61	6.69 ± 0.81	< 0.001^**‡**^	< 0.001^**†**^	< 0.001*
	Δ1℃	4.13 ± 0.74	5.12 ± 0.71	6.75 ± 0.87			
	Δ2℃	4.56 ± 0.89	6.46 ± 0.99	8.33 ± 0.84			
Trailing	Con	2.43 ± 0.57	2.88 ± 0.47	4.09 ± 0.61	< 0.001^**‡**^	< 0.001^**†**^	0.031*
	Δ1℃	2.61 ± 0.66	2.92 ± 0.49	4.15 ± 0.65			
	Δ2℃	2.84 ± 0.51	3.73 ± 0.68	5.15 ± 0.54			
Pectineus							
Leading	Con	2.08 ± 0.65	2.33 ± 0.90	2.17 ± 0.96	0.652	0.196	0.951
	Δ1℃	2.29 ± 0.98	2.32 ± 0.82	2.23 ± 0.76			
	Δ2℃	2.41 ± 0.89	2.57 ± 0.89	2.61 ± 0.86			
Trailing	Con	2.84 ± 0.49	2.95 ± 0.92	3.18 ± 1.15	0.250	0.103	0.989
	Δ1℃	3.04 ± 0.71	3.11 ± 1.27	3.27 ± 1.36			
	Δ2℃	3.18 ± 0.75	3.27 ± 0.89	3.62 ± 1.20			
Gemellus inferior							
Leading	Con	1.31 ± 0.34	1.36 ± 0.35	1.41 ± 0.41	0.153	0.627	0.977
	Δ1℃	1.29 ± 0.30	1.41 ± 0.34	1.42 ± 0.28			
	Δ2℃	1.39 ± 0.39	1.43 ± 0.30	1.48 ± 0.31			
Trailing	Con	0.78 ± 0.12	0.84 ± 0.11	0.94 ± 0.18	< 0.001^**‡**^	< 0.001^**†**^	< 0.001*
	Δ1℃	0.83 ± 0.14	0.85 ± 0.11	0.96 ± 0.18			
	Δ2℃	0.89 ± 0.17	1.10 ± 0.17	1.31 ± 0.24			
Gemellus superior							
Leading	Con	1.12 ± 0.28	1.17 ± 0.28	1.19 ± 0.38	0.255	0.677	0.977
	Δ1℃	1.15 ± 0.22	1.20 ± 0.31	1.21 ± 0.21			
	Δ2℃	1.21 ± 0.21	1.21 ± 0.26	1.27 ± 0.28			
Trailing	Con	0.71 ± 0.12	0.73 ± 0.15	0.82 ± 0.12	< 0.001^**‡**^	< 0.001^**†**^	0.004*
	Δ1℃	0.76 ± 0.17	0.77 ± 0.17	0.85 ± 0.15			
	Δ2℃	0.82 ± 0.18	0.91 ± 0.20	1.12 ± 0.21			
Gluteus Maximus							
Leading	Con	5.18 ± 0.54	4.94 ± 0.68	5.16 ± 0.81	< 0.001^**‡**^	< 0.001^**†**^	0.001*
	Δ1℃	5.29 ± 0.48	5.35 ± 0.73	5.65 ± 0.96			
	Δ2℃	5.67 ± 0.68	6.38 ± 0.70	6.86 ± 0.98			
Trailing	Con	3.26 ± 0.61	3.35 ± 0.49	3.63 ± 0.56	< 0.001^**‡**^	< 0.001^**†**^	0.008*
	Δ1℃	3.35 ± 0.66	3.39 ± 0.54	3.68 ± 0.61			
	Δ2℃	3.55 ± 0.58	4.19 ± 0.67	4.64 ± 0.67			
Piriformis							
Leading	Con	6.18 ± 0.99	6.17 ± 0.65	6.63 ± 0.86	< 0.001^**‡**^	< 0.001^**†**^	0.001*
	Δ1℃	6.11 ± 0.83	6.38 ± 0.62	7.11 ± 0.79			
	Δ2℃	6.43 ± 0.96	7.25 ± 0.91	8.42 ± 0.96			
Trailing	Con	4.43 ± 0.77	4.72 ± 0.67	5.13 ± 0.79	< 0.001^**‡**^	< 0.001^**†**^	0.024*
	Δ1℃	4.61 ± 0.66	4.75 ± 0.68	5.20 ± 0.80			
	Δ2℃	4.89 ± 0.91	5.64 ± 0.75	6.43 ± 0.91			
Vastus lateralis							
Leading	Con	0.27 ± 0.22	0.19 ± 0.23	0.17 ± 0.31	0.066	0.683	0.986
	Δ1℃	0.29 ± 0.32	0.21 ± 0.26	0.18 ± 0.28			
	Δ2℃	0.34 ± 0.30	0.26 ± 0.33	0.21 ± 0.25			
Trailing	Con	1.14 ± 0.36	1.11 ± 0.43	1.08 ± 0.35	0.498	0.727	0.998
	Δ1℃	1.16 ± 0.45	1.15 ± 0.42	1.08 ± 0.31			
	Δ2℃	1.22 ± 0.46	1.2 ± 0.41	1.15 ± 0.51			
Vastus Medialis							
Leading	Con	0.21 ± 0.22	0.14 ± 0.18	0.15 ± 0.29	0.102	0.645	0.929
	Δ1℃	0.23 ± 0.20	0.17 ± 0.21	0.15 ± 0.25			
	Δ2℃	0.29 ± 0.33	0.21 ± 0.27	0.17 ± 0.25			
Trailing	Con	0.96 ± 0.36	0.92 ± 0.34	0.89 ± 0.43	0.248	0.135	0.962
	Δ1℃	1.01 ± 0.28	0.98 ± 0.41	0.92 ± 0.36			
	Δ2℃	1.16 ± 0.37	1.12 ± 0.48	0.99 ± 0.37			
Vastus Intermedius							
Leading	Con	0.17 ± 0.14	0.13 ± 0.16	0.14 ± 0.27	0.520	0.793	0.976
	Δ1℃	0.17 ± 0.18	0.14 ± 0.17	0.15 ± 0.28			
	Δ2℃	0.20 ± 0.17	0.18 ± 0.22	0.17 ± 0.29			
Trailing	Con	1.02 ± 0.27	0.99 ± 0.35	0.99 ± 0.36	0.291	0.381	0.869
	Δ1℃	1.07 ± 0.36	1.01 ± 0.39	1.01 ± 0.35			
	Δ2℃	1.18 ± 0.52	1.18 ± 0.45	1.10 ± 0.38			
Rectus Femoris							
Leading	Con	4.89 ± 0.62	5.00 ± 0.52	5.19 ± 0.69	< 0.001^**‡**^	< 0.001^**†**^	< 0.001*
	Δ1℃	4.96 ± 0.84	5.11 ± 0.66	5.42 ± 0.73			
	Δ2℃	5.31 ± 0.74	6.35 ± 0.77	7.37 ± 1.01			
Trailing	Con	3.94 ± 0.55	4.11 ± 0.64	4.51 ± 0.54	< 0.001^**‡**^	< 0.001^**†**^	0.034*
	Δ1℃	4.12 ± 0.66	4.14 ± 0.63	4.60 ± 0.53			
	Δ2℃	4.38 ± 0.68	5.18 ± 0.57	5.68 ± 0.52			
Semitendinosus							
Leading	Con	10.67 ± 1.66	9.77 ± 1.42	9.26 ± 1.37	< 0.001^**‡**^	0.002^**†**^	0.037*
	Δ1℃	11.43 ± 1.73	10.03 ± 1.41	9.53 ± 1.29			
	Δ2℃	11.68 ± 1.42	11.36 ± 1.37	11.21 ± 1.41			
Trailing	Con	11.84 ± 1.67	11.78 ± 2.47	11.27 ± 2.30	0.106	0.686	0.988
	Δ1℃	11.91 ± 2.36	11.81 ± 2.15	11.37 ± 2.15			
	Δ2℃	12.38 ± 2.54	12.13 ± 1.77	11.94 ± 2.35			
Semimembranosus							
Leading	Con	19.91 ± 1.26	17.69 ± 1.09	16.84 ± 0.98	< 0.001^**‡**^	< 0.001^**†**^	0.048*
	Δ1℃	20.11 ± 1.16	18.11 ± 1.11	17.19 ± 1.01			
	Δ2℃	21.00 ± 1.14	20.05 ± 1.19	18.59 ± 0.98			
Trailing	Con	19.88 ± 1.96	18.35 ± 0.91	17.84 ± 0.96	< 0.001^**‡**^	< 0.001^**†**^	0.042*
	Δ1℃	20.02 ± 1.56	18.43 ± 0.96	17.92 ± 1.02			
	Δ2℃	20.64 ± 1.58	20.34 ± 1.04	19.82 ± 1.22			
Biceps femoris long head							
Leading	Con	12.57 ± 1.62	10.89 ± 0.98	10.25 ± 0.95	< 0.001^**‡**^	< 0.001^**†**^	0.003*
	Δ1℃	12.89 ± 1.22	11.06 ± 0.97	10.60 ± 0.92			
	Δ2℃	13.71 ± 1.09	13.28 ± 1.05	12.98 ± 0.96			
Trailing	Con	10.05 ± 0.96	8.70 ± 0.57	8.43 ± 0.66	< 0.001^**‡**^	< 0.001^**†**^	0.017*
	Δ1℃	10.18 ± 0.85	8.76 ± 0.59	8.48 ± 0.67			
	Δ2℃	10.67 ± 0.88	10.33 ± 0.62	9.87 ± 0.73			
Biceps Femoris short head							
Leading	Con	14.33 ± 1.08	15.07 ± 0.98	16.58 ± 1.09	< 0.001^**‡**^	< 0.001^**†**^	< 0.001*
	Δ1℃	14.51 ± 1.12	15.21 ± 1.06	16.82 ± 1.23			
	Δ2℃	14.69 ± 1.50	17.01 ± 0.94	19.83 ± 1.17			
Trailing	Con	19.03 ± 4.37	18.38 ± 2.96	18.28 ± 4.07	0.405	0.580	0.975
	Δ1℃	19.22 ± 3.01	18.95 ± 2.61	18.84 ± 4.89			
	Δ2℃	20.36 ± 4.84	19.51 ± 3.11	19.05 ± 5.84			
Sartorius							
Leading	Con	3.82 ± 0.86	3.92 ± 0.68	4.70 ± 0.67	< 0.001^**‡**^	< 0.001^**†**^	< 0.001*
	Δ1℃	4.01 ± 0.95	4.03 ± 0.69	4.82 ± 0.71			
	Δ2℃	4.12 ± 0.87	4.82 ± 0.95	6.59 ± 0.98			
Trailing	Con	3.63 ± 0.52	3.81 ± 0.39	4.25 ± 0.41	< 0.001^**‡**^	< 0.001^**†**^	< 0.001*
	Δ1℃	3.67 ± 0.45	3.84 ± 0.41	4.29 ± 0.38			
	Δ2℃	3.91 ± 0.53	4.66 ± 0.51	5.24 ± 0.51			
Gracilis muscle							
Leading	Con	2.58 ± 0.70	2.59 ± 0.70	2.76 ± 0.84	0.141	0.489	0.952
	Δ1℃	2.75 ± 0.78	2.82 ± 0.92	2.99 ± 1.55			
	Δ2℃	2.80 ± 0.88	3.01 ± 0.93	3.12 ± 1.24			
Trailing	Con	2.93 ± 0.73	2.82 ± 0.59	2.71 ± 0.74	0.053	0.613	0.998
	Δ1℃	3.02 ± 0.62	2.93 ± 0.86	2.75 ± 0.75			
	Δ2℃	3.15 ± 0.63	3.06 ± 0.81	2.86 ± 1.01			
Tensor Fasciae Lata							
Leading	Con	4.53 ± 0.69	4.70 ± 0.67	5.23 ± 0.59	< 0.001^**‡**^	< 0.001^**†**^	0.032*
	Δ1℃	4.76 ± 0.77	4.80 ± 0.69	5.39 ± 0.76			
	Δ2℃	5.01 ± 0.97	5.34 ± 0.79	6.51 ± 0.71			
Trailing	Con	6.90 ± 1.73	7.09 ± 1.72	7.23 ± 1.46	0.319	0.619	0.988
	Δ1℃	7.21 ± 1.56	7.43 ± 1.60	7.51 ± 2.34			
	Δ2℃	7.39 ± 2.14	7.53 ± 2.42	7.92 ± 2.43			
Soleus							
Leading	Con	0.01 ± 0.02	0.01 ± 0.01	0.01 ± 0.02	0.057	0.421	0.477
	Δ1℃	0.03 ± 0.09	0.02 ± 0.06	0.01 ± 0.02			
	Δ2℃	0.04 ± 0.11	0.02 ± 0.05	0.01 ± 0.01			
Trailing	Con	0.07 ± 0.07	0.08 ± 0.12	0.09 ± 0.11	0.346	0.411	0.682
	Δ1℃	0.08 ± 0.12	0.09 ± 0.11	0.09 ± 0.15			
	Δ2℃	0.08 ± 0.09	0.11 ± 0.14	0.18 ± 0.37			
Gastrocnemius							
Leading	Con	1.26 ± 0.51	1.28 ± 0.42	1.37 ± 0.61	0.051	0.191	0.766
	Δ1℃	1.27 ± 0.49	1.29 ± 0.54	1.45 ± 0.78			
	Δ2℃	1.41 ± 0.37	1.60 ± 0.62	1.71 ± 0.71			
Trailing	Con	3.09 ± 1.12	3.31 ± 1.01	3.41 ± 2.01	0.442	0.682	0.999
	Δ1℃	3.21 ± 1.45	3.36 ± 1.53	3.41 ± 2.08			
	Δ2℃	3.41 ± 0.83	3.65 ± 1.73	3.77 ± 1.93			
Flexor digitorum longus							
Leading	Con	1.20 ± 1.97	1.17 ± 1.01	1.12 ± 2.13	0.602	0.786	0.958
	Δ1℃	1.47 ± 2.01	1.37 ± 1.41	1.30 ± 1.64			
	Δ2℃	1.82 ± 2.34	1.39 ± 1.57	1.31 ± 2.21			
Trailing	Con	0.79 ± 0.64	0.82 ± 0.54	0.84 ± 0.82	0.796	0.807	0.999
	Δ1℃	0.82 ± 0.84	0.86 ± 0.99	0.95 ± 0.67			
	Δ2℃	0.93 ± 0.99	0.97 ± 2.07	1.07 ± 1.10			
Flexor hallucis longus							
Leading	Con	0.01 ± 0.03	0.01 ± 0.01	0.01 ± 0.04	0.302	0.928	0.981
	Δ1℃	0.01 ± 0.02	0.01 ± 0.02	0.01 ± 0.01			
	Δ2℃	0.01 ± 0.02	0.01 ± 0.02	0.01 ± 0.02			
Trailing	Con	0.05 ± 0.06	0.05 ± 0.05	0.06 ± 0.08	0.806	0.710	0.999
	Δ1℃	0.06 ± 0.05	0.06 ± 0.05	0.07 ± 0.12			
	Δ2℃	0.07 ± 0.07	0.07 ± 0.04	0.08 ± 0.22			
Tibialis posterior							
Leading	Con	2.69 ± 4.78	2.28 ± 3.83	2.04 ± 3.55	0.678	0.588	0.385
	Δ1℃	2.88 ± 3.58	2.38 ± 3.55	2.15 ± 3.10			
	Δ2℃	3.16 ± 3.42	2.69 ± 3.89	2.27 ± 3.04			
Trailing	Con	2.89 ± 2.12	2.75 ± 2.43	2.32 ± 1.98	0.252	0.833	0.998
	Δ1℃	3.22 ± 3.55	3.04 ± 4.06	2.43 ± 2.95			
	Δ2℃	3.44 ± 3.64	3.09 ± 1.98	2.79 ± 1.99			
Peroneus brevis							
Leading	Con	10.24 ± 11.89	11.03 ± 11.65	12.76 ± 11.48	0.233	0.929	0.999
	Δ1℃	10.61 ± 12.71	11.50 ± 14.53	13.13 ± 10.78			
	Δ2℃	11.38 ± 12.37	11.79 ± 12.31	14.62 ± 11.99			
Trailing	Con	11.34 ± 7.39	12.21 ± 11.08	15.15 ± 9.63	0.118	0.821	1.000
	Δ1℃	12.45 ± 9.89	12.6 ± 14.54	15.8 ± 10.95			
	Δ2℃	13.15 ± 10.63	14.22 ± 11.4	16.7 ± 15.95			
Tibialis Anterior							
Leading	Con	5.11 ± 5.10	5.07 ± 5.55	4.59 ± 5.89	0.112	0.793	0.91
	Δ1℃	5.91 ± 5.49	5.65 ± 5.86	4.72 ± 5.32			
	Δ2℃	6.81 ± 5.97	6.63 ± 6.90	4.94 ± 6.82			
Trailing	Con	8.05 ± 5.69	8.30 ± 5.17	8.62 ± 5.06	0.708	0.693	0.925
	Δ1℃	8.22 ± 2.67	8.48 ± 5.31	8.82 ± 5.89			
	Δ2℃	9.18 ± 3.05	9.43 ± 5.38	9.75 ± 6.75			
Extensor Digitorum Longus							
Leading	Con	3.31 ± 1.13	3.35 ± 1.24	3.52 ± 1.62	0.103	0.067	0.255
	Δ1℃	3.46 ± 1.50	3.53 ± 1.71	3.78 ± 1.91			
	Δ2℃	4.21 ± 1.83	5.33 ± 2.07	6.94 ± 1.86			
Trailing	Con	7.63 ± 3.56	8.14 ± 4.21	8.31 ± 4.34	0.563	0.840	1.000
	Δ1℃	8.04 ± 4.14	8.40 ± 4.81	8.68 ± 4.11			
	Δ2℃	8.41 ± 6.16	9.00 ± 3.34	9.12 ± 8.06			
Extensor hallucis longus							
Leading	Con	0.68 ± 0.28	0.69 ± 0.35	0.73 ± 0.47	0.497	0.302	0.984
	Δ1℃	0.73 ± 0.31	0.75 ± 0.34	0.80 ± 0.48			
	Δ2℃	0.81 ± 0.29	0.83 ± 0.45	0.92 ± 0.66			
Trailing	Con	2.81 ± 1.14	2.64 ± 1.29	2.49 ± 1.71	0.153	0.596	0.995
	Δ1℃	2.98 ± 1.08	2.72 ± 0.66	2.65 ± 1.12			
	Δ2℃	3.11 ± 1.42	3.02 ± 0.76	2.83 ± 1.30			

“‡” Main effect of height (*p* < 0.05).

“†” Main effect of T_Oral_ (*p* < 0.05).

“*” Significant T_Oral_ x height interaction effects (*p* < 0.05).

**Table 3:** Muscle activation when leading (T3) and trailing (T1) limbs heel-strike event at three T_oral_ (Con, Δ1℃, Δ2℃) and heights (10%, 20%,30%).

Characteristic	Treatment	Obstacle height(10%LL)	Obstacle height(20%LL)	Obstacle height(30%LL)	*p-*values		
					Main effects (Height)	Main effects (T_Oral_)	Interaction (T_Oral_ x Height)
Gluteus Minimus							
Leading	Con	3.35 ± 0.38	3.46 ± 0.29	3.70 ± 0.64	< 0.001^**‡**^	0.001^**†**^	0.037*
	Δ1℃	3.55 ± 0.44	3.59 ± 0.36	3.67 ± 0.64			
	Δ2℃	3.70 ± 0.64	4.06 ± 0.48	4.41 ± 0.68			
Trailing	Con	3.82 ± 0.5	3.95 ± 0.4	4.04 ± 0.47	< 0.001^**‡**^	< 0.001^**†**^	0.025*
	Δ1℃	3.85 ± 0.59	3.99 ± 0.41	4.10 ± 0.47			
	Δ2℃	4.07 ± 0.62	4.87 ± 0.48	5.05 ± 0.55			
Gluteus Medius							
Leading	Con	5.91 ± 0.57	5.09 ± 0.52	4.52 ± 0.75	< 0.001^**‡**^	< 0.001^**†**^	0.018*
	Δ1℃	5.92 ± 0.55	5.16 ± 0.57	4.62 ± 0.77			
	Δ2℃	6.15 ± 0.59	5.91 ± 0.58	5.60 ± 0.77			
Trailing	Con	10.55 ± 0.80	10.85 ± 0.59	11.31 ± 0.57	< 0.001^**‡**^	< 0.001^**†**^	0.018*
	Δ1℃	10.66 ± 0.83	10.95 ± 0.61	11.36 ± 0.59			
	Δ2℃	11.16 ± 0.93	11.84 ± 0.75	12.89 ± 0.71			
Adductor Longus							
Leading	Con	1.46 ± 0.62	1.71 ± 0.36	1.93 ± 0.40	< 0.001^**‡**^	< 0.001^**†**^	0.030*
	Δ1℃	1.64 ± 0.71	1.77 ± 0.36	1.99 ± 0.39			
	Δ2℃	1.87 ± 0.59	2.21 ± 0.55	2.75 ± 0.36			
Trailing	Con	0.61 ± 0.22	0.82 ± 0.09	0.88 ± 0.11	< 0.001^**‡**^	< 0.001^**†**^	< 0.001*
	Δ1℃	0.69 ± 0.27	0.84 ± 0.11	0.91 ± 0.11			
	Δ2℃	0.76 ± 0.25	1.03 ± 0.17	1.42 ± 0.25			
Adductor Magnus							
Leading	Con	1.73 ± 0.7	1.76 ± 0.37	1.8 ± 0.38	0.011^**‡**^	0.001^**†**^	0.034*
	Δ1℃	1.8 ± 0.44	1.81 ± 0.40	1.85 ± 0.43			
	Δ2℃	1.96 ± 0.60	2.31 ± 0.61	2.52 ± 0.45			
Trailing	Con	0.83 ± 0.27	0.85 ± 0.13	0.96 ± 0.17	< 0.001^**‡**^	< 0.001^**†**^	0.037*
	Δ1℃	0.85 ± 0.29	0.87 ± 0.13	0.98 ± 0.18			
	Δ2℃	0.97 ± 0.22	1.19 ± 0.27	1.39 ± 0.32			
Quadratus Femoris							
Leading	Con	2.47 ± 0.52	2.56 ± 0.51	2.88 ± 0.50	< 0.001^**‡**^	0.001^**†**^	0.007*
	Δ1℃	2.56 ± 0.48	2.61 ± 0.51	2.97 ± 0.51			
	Δ2℃	2.78 ± 0.57	3.12 ± 0.60	3.73 ± 0.57			
Trailing	Con	1.13 ± 0.27	1.23 ± 0.31	1.62 ± 0.31	< 0.001^**‡**^	< 0.001^**†**^	< 0.001*
	Δ1℃	1.18 ± 0.28	1.27 ± 0.35	1.64 ± 0.32			
	Δ2℃	1.27 ± 0.28	1.81 ± 0.35	2.43 ± 0.48			
Adductor Brevis							
Leading	Con	1.13 ± 0.46	1.31 ± 0.49	1.43 ± 0.43	< 0.001^**‡**^	0.006^**†**^	0.023*
	Δ1℃	1.23 ± 0.44	1.37 ± 0.54	1.49 ± 0.46			
	Δ2℃	1.39 ± 0.57	1.66 ± 0.61	2.13 ± 0.44			
Trailing	Con	0.31 ± 0.13	0.42 ± 0.07	0.59 ± 0.11	< 0.001^**‡**^	< 0.001^**†**^	< 0.001*
	Δ1℃	0.34 ± 0.16	0.43 ± 0.07	0.61 ± 0.13			
	Δ2℃	0.41 ± 0.14	0.66 ± 0.12	1.04 ± 0.20			
Obturator Internus Muscle							
Leading	Con	2.78 ± 0.38	2.96 ± 0.44	3.00 ± 0.42	< 0.001^**‡**^	< 0.001^**†**^	0.049*
	Δ1℃	2.94 ± 0.45	3.01 ± 0.43	3.05 ± 0.42			
	Δ2℃	3.05 ± 0.50	3.40 ± 0.47	3.74 ± 0.41			
Trailing	Con	2.28 ± 0.29	2.35 ± 0.21	2.57 ± 0.33	< 0.001^**‡**^	< 0.001^**†**^	< 0.001*
	Δ1℃	2.32 ± 0.31	2.37 ± 0.20	2.61 ± 0.32			
	Δ2℃	2.43 ± 0.41	3.19 ± 0.36	3.63 ± 0.54			
Obturator Externus Muscle							
Leading	Con	3.86 ± 0.56	3.86 ± 0.56	3.86 ± 0.56	< 0.001^**‡**^	< 0.001^**†**^	0.011*
	Δ1℃	3.92 ± 0.51	4.52 ± 0.62	5.47 ± 0.57			
	Δ2℃	4.08 ± 0.56	5.23 ± 0.68	6.45 ± 0.65			
Trailing	Con	1.94 ± 0.64	2.12 ± 0.44	2.61 ± 0.57	< 0.001^**‡**^	< 0.001^**†**^	0.005*
	Δ1℃	1.95 ± 0.71	2.13 ± 0.48	2.68 ± 0.67			
	Δ2℃	2.21 ± 0.49	2.87 ± 0.56	3.82 ± 0.82			
Pectineus							
Leading	Con	3.36 ± 0.54	3.52 ± 0.51	3.80 ± 0.52	< 0.001^**‡**^	< 0.001^**†**^	< 0.001*
	Δ1℃	3.43 ± 0.65	3.58 ± 0.51	3.86 ± 0.56			
	Δ2℃	3.61 ± 0.62	4.62 ± 0.57	5.35 ± 0.71			
Trailing	Con	1.83 ± 0.62	1.89 ± 0.41	2.26 ± 0.49	< 0.001^**‡**^	< 0.001^**†**^	0.046*
	Δ1℃	1.92 ± 0.75	1.91 ± 0.41	2.31 ± 0.49			
	Δ2℃	2.12 ± 0.76	2.69 ± 0.60	3.26 ± 0.53			
Gemellus Inferior							
Leading	Con	0.91 ± 0.12	0.98 ± 0.20	1.00 ± 0.17	0.105	0.609	0.974
	Δ1℃	0.95 ± 0.17	0.99 ± 0.25	1.01 ± 0.22			
	Δ2℃	0.99 ± 0.26	1.01 ± 0.17	1.04 ± 0.17			
Trailing	Con	0.56 ± 0.09	0.59 ± 0.11	0.61 ± 0.12	< 0.001^**‡**^	< 0.001^**†**^	< 0.001*
	Δ1℃	0.58 ± 0.09	0.60 ± 0.12	0.62 ± 0.14			
	Δ2℃	0.61 ± 0.11	0.79 ± 0.11	0.89 ± 0.18			
Gemellus Superior							
Leading	Con	0.81 ± 0.15	0.87 ± 0.23	0.87 ± 0.14	0.067	0.446	0.965
	Δ1℃	0.85 ± 0.14	0.89 ± 0.19	0.89 ± 0.12			
	Δ2℃	0.89 ± 0.21	0.91 ± 0.17	0.94 ± 0.09			
Trailing	Con	0.47 ± 0.06	0.48 ± 0.06	0.52 ± 0.08	< 0.001^**‡**^	< 0.001^**†**^	< 0.001*
	Δ1℃	0.48 ± 0.08	0.49 ± 0.07	0.53 ± 0.09			
	Δ2℃	0.52 ± 0.09	0.66 ± 0.11	0.74 ± 0.17			
Gluteus Maximus							
Leading	Con	4.78 ± 0.33	4.92 ± 0.46	4.93 ± 0.51	< 0.001^**‡**^	< 0.001^**†**^	0.001*
	Δ1℃	4.87 ± 0.40	5.00 ± 0.48	5.07 ± 0.56			
	Δ2℃	4.96 ± 0.41	5.59 ± 0.58	5.85 ± 0.65			
Trailing	Con	3.85 ± 0.65	3.13 ± 0.33	3.01 ± 0.41	< 0.001^**‡**^	< 0.001^**†**^	0.013*
	Δ1℃	3.96 ± 0.77	3.16 ± 0.36	3.04 ± 0.42			
	Δ2℃	4.05 ± 0.69	3.93 ± 0.51	3.70 ± 0.65			
*Piriformis*							
Leading	Con	4.72 ± 0.71	4.99 ± 0.57	5.71 ± 0.41	< 0.001^**‡**^	< 0.001^**†**^	0.001*
	Δ1℃	4.94 ± 0.76	5.08 ± 0.65	5.85 ± 0.52			
	Δ2℃	5.1 ± 0.72	5.77 ± 0.63	6.94 ± 0.64			
Trailing	Con	2.69 ± 0.55	2.72 ± 0.37	2.81 ± 0.41	0.001^**‡**^	< 0.001^**†**^	0.001*
	Δ1℃	2.77 ± 0.54	2.79 ± 0.35	2.83 ± 0.41			
	Δ2℃	2.93 ± 0.66	3.63 ± 0.67	3.92 ± 0.52			
Vastus Lateralis							
Leading	Con	1 ± 0.1	0.54 ± 0.11	0.29 ± 0.09	< 0.001^**‡**^	< 0.001^**†**^	0.009*
	Δ1℃	1.03 ± 0.12	0.57 ± 0.11	0.3 ± 0.09			
	Δ2℃	1.09 ± 0.16	0.80 ± 0.10	0.44 ± 0.09			
Trailing	Con	4.83 ± 0.92	5.03 ± 0.83	5.09 ± 1.17	0.490	0.490	0.998
	Δ1℃	5.00 ± 1.02	5.15 ± 1.19	5.14 ± 1.26			
	Δ2℃	5.07 ± 1.11	5.36 ± 1.23	5.34 ± 1.23			
Vastus Medialis							
Leading	Con	0.86 ± 0.11	0.49 ± 0.04	0.22 ± 0.03	< 0.001^**‡**^	< 0.001^**†**^	0.045*
	Δ1℃	0.87 ± 0.11	0.51 ± 0.04	0.23 ± 0.04			
	Δ2℃	0.93 ± 0.1	0.66 ± 0.04	0.37 ± 0.04			
Trailing	Con	4.18 ± 1.27	4.44 ± 0.94	4.64 ± 0.98	0.057	0.481	0.987
	Δ1℃	4.26 ± 0.91	4.54 ± 0.96	4.73 ± 1.25			
	Δ2℃	4.47 ± 1.04	4.77 ± 1.65	4.94 ± 0.96			
Vastus Intermedius							
Leading	Con	0.81 ± 0.11	0.35 ± 0.03	0.24 ± 0.02	< 0.001^**‡**^	< 0.001^**†**^	0.009*
	Δ1℃	0.81 ± 0.11	0.36 ± 0.04	0.25 ± 0.03			
	Δ2℃	0.85 ± 0.13	0.54 ± 0.04	0.36 ± 0.04			
Trailing	Con	4.10 ± 1.59	4.26 ± 1.21	4.51 ± 0.94	0.059	0.868	0.946
	Δ1℃	4.19 ± 0.88	4.34 ± 0.87	4.63 ± 0.89			
	Δ2℃	4.20 ± 0.94	4.39 ± 1.33	4.67 ± 0.79			
Rectus Femoris							
Leading	Con	7.21 ± 0.67	6.43 ± 0.72	5.99 ± 0.68	< 0.001^**‡**^	< 0.001^**†**^	0.042*
	Δ1℃	7.26 ± 0.77	6.52 ± 0.68	6.08 ± 0.72			
	Δ2℃	7.57 ± 0.87	7.39 ± 0.69	7.12 ± 0.76			
Trailing	Con	9.30 ± 1.52	9.33 ± 1.71	9.46 ± 1.96	0.489	0.850	0.979
	Δ1℃	9.38 ± 2.20	9.53 ± 2.14	9.70 ± 2.70			
	Δ2℃	9.46 ± 2.12	9.60 ± 1.59	9.97 ± 2.52			
Semitendinosus							
Leading	Con	6.45 ± 0.71	6.56 ± 0.60	7.21 ± 0.77	< 0.001^**‡**^	0.002^**†**^	0.031*
	Δ1℃	6.64 ± 0.77	6.69 ± 0.59	7.31 ± 0.85			
	Δ2℃	6.73 ± 0.77	7.37 ± 0.63	8.19 ± 0.83			
Trailing	Con	7.95 ± 1.29	8.16 ± 1.43	8.32 ± 1.93	0.217	0.951	0.997
	Δ1℃	8.01 ± 1.57	8.20 ± 1.98	8.36 ± 2.62			
	Δ2℃	8.19 ± 1.93	8.36 ± 1.99	8.42 ± 2.14			
Semimembranosus							
Leading	Con	12.07 ± 0.72	12.14 ± 0.57	12.79 ± 0.68	< 0.001^**‡**^	< 0.001^**†**^	0.012*
	Δ1℃	12.44 ± 0.86	12.3 ± 0.62	12.86 ± 0.7			
	Δ2℃	12.49 ± 0.81	13.35 ± 0.76	13.96 ± 0.77			
Trailing	Con	13.87 ± 2.31	14.14 ± 2.69	14.42 ± 1.94	0.219	0.946	0.954
	Δ1℃	14.03 ± 2.18	14.27 ± 2.38	14.55 ± 3.27			
	Δ2℃	14.11 ± 2.18	14.33 ± 2.06	14.66 ± 1.98			
Biceps Femoris long head							
Leading	Con	9.31 ± 0.74	9.27 ± 0.66	9.65 ± 0.73	< 0.001^**‡**^	< 0.001^**†**^	0.004*
	Δ1℃	9.35 ± 0.73	9.36 ± 0.71	9.79 ± 0.77			
	Δ2℃	9.51 ± 0.85	10.24 ± 0.76	11.02 ± 0.88			
Trailing	Con	6.45 ± 1.17	6.55 ± 0.86	6.56 ± 1.65	0.605	0.286	0.995
	Δ1℃	6.55 ± 0.86	6.74 ± 0.69	6.77 ± 1.13			
	Δ2℃	6.91 ± 1.05	6.98 ± 1.19	7.08 ± 1.79			
Biceps Femoris short head							
Leading	Con	6.57 ± 1.04	7.19 ± 0.67	10.26 ± 0.87	< 0.001^**‡**^	< 0.001^**†**^	0.024*
	Δ1℃	6.66 ± 0.85	7.27 ± 0.67	10.33 ± 0.87			
	Δ2℃	6.98 ± 0.94	8.39 ± 0.76	11.72 ± 0.86			
Trailing	Con	8.88 ± 2.16	9.27 ± 1.49	9.62 ± 1.96	0.069	0.768	0.995
	Δ1℃	9.11 ± 2.72	9.44 ± 3.47	9.89 ± 4.14			
	Δ2℃	9.42 ± 2.49	9.69 ± 2.49	10.36 ± 2.98			
Sartorius							
Leading	Con	3.38 ± 0.56	3.8 ± 0.55	4.89 ± 0.6	< 0.001^**‡**^	< 0.001^**†**^	0.001*
	Δ1℃	3.42 ± 0.74	3.95 ± 0.66	4.97 ± 0.67			
	Δ2℃	3.72 ± 0.71	4.88 ± 0.72	6.08 ± 0.63			
Trailing	Con	2.86 ± 0.50	2.90 ± 0.43	3.29 ± 0.55	< 0.001^**‡**^	< 0.001^**†**^	< 0.001*
	Δ1℃	2.87 ± 0.61	2.93 ± 0.45	3.32 ± 0.56			
	Δ2℃	3.11 ± 0.53	3.77 ± 0.59	4.71 ± 0.74			
Gracilis muscle							
Leading	Con	1.55 ± 0.47	1.62 ± 0.43	1.62 ± 0.43	0.350	0.192	0.918
	Δ1℃	1.60 ± 0.42	1.64 ± 0.52	1.75 ± 0.56			
	Δ2℃	1.83 ± 0.64	1.84 ± 0.54	1.89 ± 0.58			
Trailing	Con	1.29 ± 0.37	1.39 ± 0.41	1.42 ± 0.38	0.087	0.728	0.971
	Δ1℃	1.34 ± 0.39	1.42 ± 0.47	1.45 ± 0.52			
	Δ2℃	1.42 ± 0.33	1.45 ± 0.45	1.53 ± 0.54			
Tensor Fasciae Lata							
Leading	Con	4.23 ± 1.34	4.68 ± 1.44	4.87 ± 1.36	0.054	0.203	0.976
	Δ1℃	4.57 ± 1.58	4.85 ± 1.63	4.95 ± 1.66			
	Δ2℃	5.18 ± 2.47	5.43 ± 1.60	5.61 ± 1.32			
Trailing	Con	7.35 ± 1.38	7.39 ± 1.28	7.89 ± 2.04	0.055	0.858	0.994
	Δ1℃	7.38 ± 2.07	7.49 ± 1.59	7.99 ± 2.47			
	Δ2℃	7.46 ± 0.99	7.75 ± 1.36	8.13 ± 1.57			
Soleus							
Leading	Con	0.01 ± 0.05	0.01 ± 0.03	0.01 ± 0.03	0.139	0.484	0.857
	Δ1℃	0.02 ± 0.03	0.02 ± 0.05	0.01 ± 0.02			
	Δ2℃	0.03 ± 0.05	0.03 ± 0.04	0.02 ± 0.03			
Trailing	Con	2.87 ± 1.31	3.02 ± 1.14	3.21 ± 1.11	0.583	0.746	0.997
	Δ1℃	3.08 ± 1.11	3.11 ± 1.31	3.23 ± 1.37			
	Δ2℃	3.14 ± 0.94	3.24 ± 0.90	3.31 ± 1.21			
Gastrocnemius							
Leading	Con	2.01 ± 0.31	1.55 ± 0.36	1.27 ± 0.27	< 0.001^**‡**^	< 0.001^**†**^	0.018*
	Δ1℃	1.98 ± 0.38	1.62 ± 0.38	1.33 ± 0.32			
	Δ2℃	2.22 ± 0.34	2.13 ± 0.38	1.95 ± 0.28			
Trailing	Con	9.79 ± 2.49	9.61 ± 1.5	9.58 ± 1.96	0.666	0.884	0.999
	Δ1℃	9.98 ± 2.46	9.67 ± 1.72	9.63 ± 2.15			
	Δ2℃	10.03 ± 1.79	9.87 ± 1.86	9.77 ± 1.79			
Flexor digitorum longus							
Leading	Con	0.71 ± 0.97	0.66 ± 1.13	0.52 ± 0.96	0.086	0.528	0.787
	Δ1℃	0.82 ± 1.08	0.8 ± 0.98	0.63 ± 0.73			
	Δ2℃	1.24 ± 1.33	0.89 ± 0.99	0.82 ± 1.26			
Trailing	Con	3.27 ± 1.1	3.18 ± 0.81	3.13 ± 1.13	0.679	0.422	0.998
	Δ1℃	3.38 ± 1.87	3.22 ± 1.13	3.21 ± 0.95			
	Δ2℃	3.66 ± 1.95	3.51 ± 0.93	3.41 ± 1.06			
Flexor hallucis longus							
Leading	Con	0.01 ± 0.02	0.01 ± 0.01	0.01 ± 0.02	0.160	0.081	0.964
	Δ1℃	0.01 ± 0.02	0.01 ± 0.01	0.01 ± 0.01			
	Δ2℃	0.02 ± 0.03	0.01 ± 0.03	0.01 ± 0.01			
Trailing	Con	0.23 ± 0.26	0.25 ± 0.22	0.28 ± 0.31	0.562	0.744	0.976
	Δ1℃	0.25 ± 0.22	0.27 ± 0.19	0.29 ± 0.2			
	Δ2℃	0.27 ± 0.13	0.29 ± 0.13	0.31 ± 0.26			
Tibialis posterior							
Leading	Con	1.75 ± 3.06	1.6 ± 2.24	1.44 ± 2.93	0.876	0.793	0.954
	Δ1℃	1.85 ± 2.66	1.84 ± 3.48	1.63 ± 2.6			
	Δ2℃	2.11 ± 3.21	2.07 ± 2.95	1.81 ± 3.3			
Trailing	Con	6.06 ± 3.96	6.18 ± 1.85	6.21 ± 2.4	0.921	0.713	0.973
	Δ1℃	6.08 ± 2.52	6.24 ± 2.52	6.28 ± 1.39			
	Δ2℃	6.47 ± 3.48	6.58 ± 3.10	6.69 ± 1.13			
Peroneus brevis							
Leading	Con	9.01 ± 7.99	10 ± 8.78	11.15 ± 7.71	0.305	0.795	0.987
	Δ1℃	10.3 ± 8.32	11.03 ± 8.62	11.33 ± 10.3			
	Δ2℃	11.02 ± 9.12	11.28 ± 8.98	12.91 ± 8.94			
Trailing	Con	16.73 ± 4.89	17.26 ± 6.70	17.53 ± 3.23	0.764	0.717	0.946
	Δ1℃	17.11 ± 4.97	17.50 ± 7.29	17.71 ± 6.57			
	Δ2℃	17.92 ± 5.55	18.11 ± 5.43	18.66 ± 6.06			
Tibialis anterior							
Leading	Con	3.38 ± 2.78	2.93 ± 2.2	2.76 ± 2.33	0.171	0.624	0.972
	Δ1℃	3.6 ± 2.38	3.51 ± 2.92	3.02 ± 2.51			
	Δ2℃	4.07 ± 2.48	3.84 ± 3.01	3.34 ± 3.00			
Trailing	Con	4.46 ± 1.50	4.62 ± 0.89	4.78 ± 1.58	0.604	0.465	0.892
	Δ1℃	4.67 ± 1.87	4.73 ± 1.38	4.90 ± 2.11			
	Δ2℃	5.00 ± 1.57	5.10 ± 1.46	5.20 ± 1.59			
Extensor digitorum longus							
Leading	Con	2.54 ± 0.88	2.61 ± 1.22	2.78 ± 1.12	0.104	0.134	0.893
	Δ1℃	2.63 ± 0.99	2.64 ± 0.85	2.88 ± 1.12			
	Δ2℃	2.98 ± 1.24	3.05 ± 1.51	3.56 ± 1.24			
Trailing	Con	5.92 ± 1.86	6.03 ± 1.72	6.11 ± 1.95	0.924	0.874	0.874
	Δ1℃	6.07 ± 2.50	6.16 ± 3.11	6.20 ± 2.96			
	Δ2℃	6.26 ± 3.02	6.34 ± 1.72	6.40 ± 2.60			
Extensor hallucis longus							
Leading	Con	0.55 ± 0.21	0.55 ± 0.25	0.63 ± 0.32	0.427	0.043	0.985
	Δ1℃	0.59 ± 0.19	0.6 ± 0.43	0.66 ± 0.48			
	Δ2℃	0.98 ± 0.87	1.06 ± 1.29	1.16 ± 1.24			
Trailing	Con	1.34 ± 0.34	1.37 ± 0.35	1.44 ± 0.33	0.346	0.795	0.997
	Δ1℃	1.37 ± 0.58	1.45 ± 0.73	1.51 ± 0.91			
	Δ2℃	1.41 ± 0.58	1.51 ± 0.55	1.58 ± 0.97			

“‡” Main effect of height (*p* < 0.05).

“†” Main effect of T_Oral_ (*p* < 0.05).

“*” Significant T_Oral_ x height interaction effects (*p* < 0.05).

### Toe-off event of leading and trailing limbs

**Figure 2:**
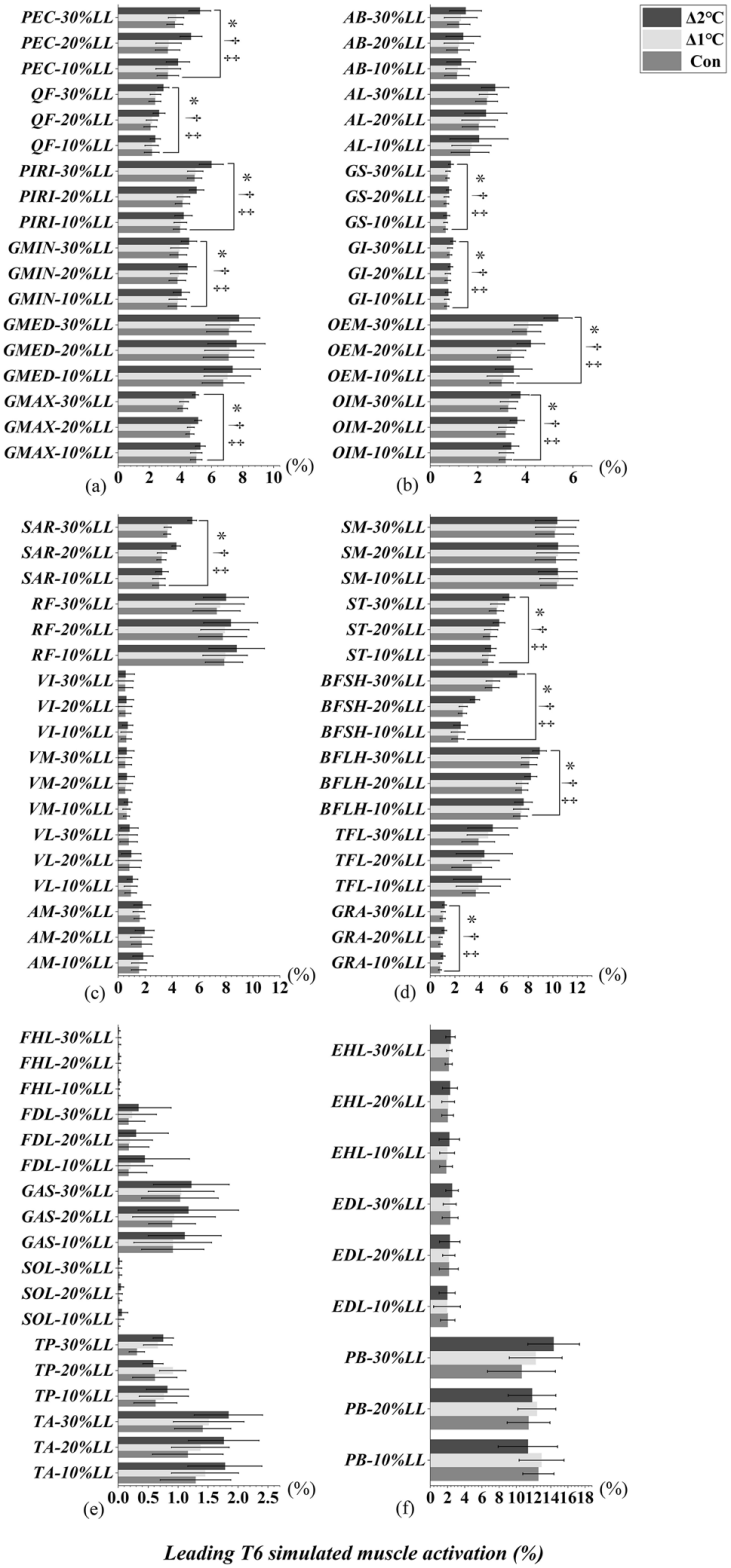
Leading limb simulated muscles activations in T6 (toe-off) event with systemic increase of T_oral_ from baseline at obstacle height of 10%, 20% and 30% of leg’s length. “*” Indicates significant T_oral_ x height interaction effects (*p* < 0.05). “†” Indicates a significant difference between Δ2℃ and Con at obstacle height of 20% and 30% leg length (*p* < 0.05). “‡” Indicates a significant difference between Δ2℃ and Δ1℃ at obstacle height of 20% and 30% leg length (*p* < 0.05).

**Figure 3:**
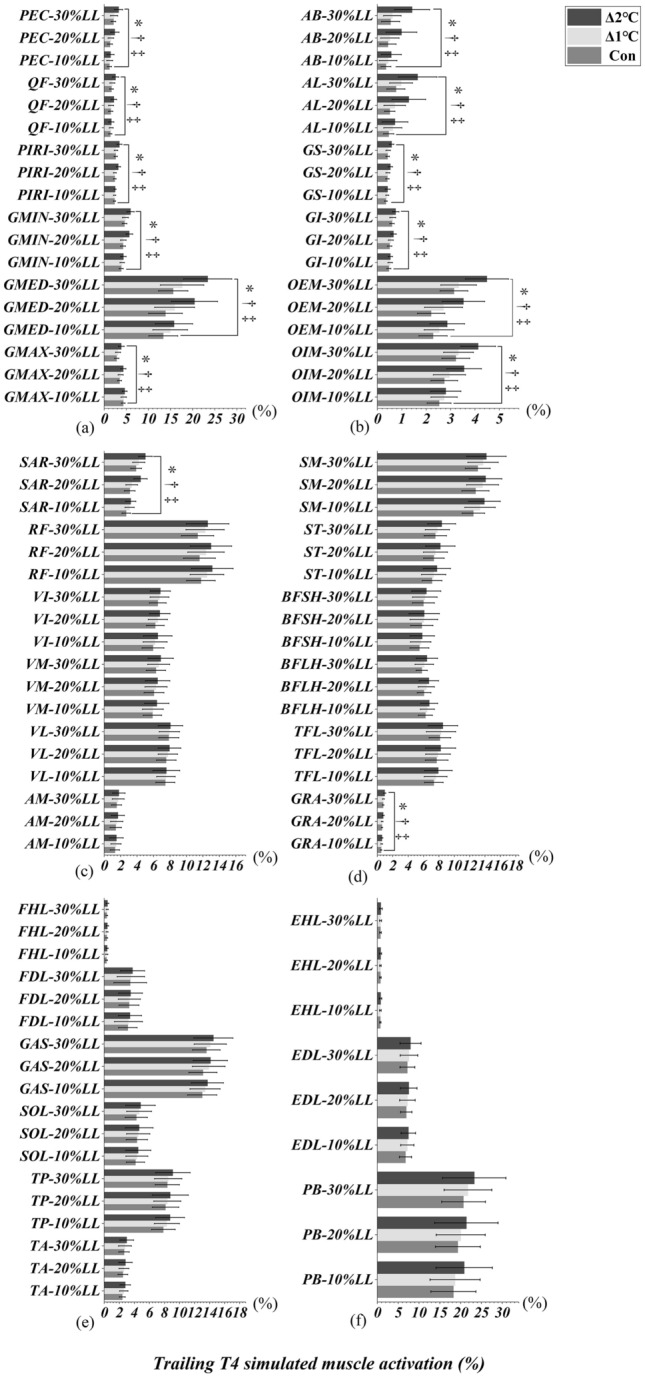
Trailing limb simulated muscles activations in T4 (toe-off) event with systemic increase of T_oral_ from baseline at obstacle height of 10%, 20% and 30% of leg’s length. “*” Indicates significant T_oral_ x height interaction effects (*p* < 0.05). “†” Indicates a significant difference between Δ2℃ and Con at obstacle height of 20% and 30% leg length (*p* < 0.05). “‡” Indicates a significant difference between Δ2℃ and Δ1℃ at obstacle height of 20% and 30% leg length (*p* < 0.05).

Significant interactions between T_oral_ and obstacle heights were observed in PEC, QF, PIRI, GMIN, GMAX, GS, GI, OEM, OIM, SAR and GRA when leading limb (Table 1, Fig. 2) and trailing limb (Table 1, Fig. 3) were in the toe-off event (T6/T4, All *p* < 0.05). ST, BFSH, and BFLH only interacted when the leading limb was in the toe-off event (All *p* < 0.034, Table 1, Fig. 2d). GMED, AB, and AL only interacted when the trailing limb was in the toe-off event (All *p* < 0.05, Table 1, Fig. 3a-b). Furthermore, the simple main effect showed that the lower limb simulated muscle activation were greater in Δ2°C than in Δ1℃ and Con when both leading and trailing limbs were toe-off event to cross an obstacle height of 20% and 30% leg length (All *p* < 0.001, effect size varying from 0.61 to 6.47, Figs. 2, 3). However, the above simulated muscle activation were not different between Δ1°C and Con (All *p* > 0.05, Figs. 2, 3).

### Toe-above-obstacle event of leading and trailing limbs

**Figure 4:**
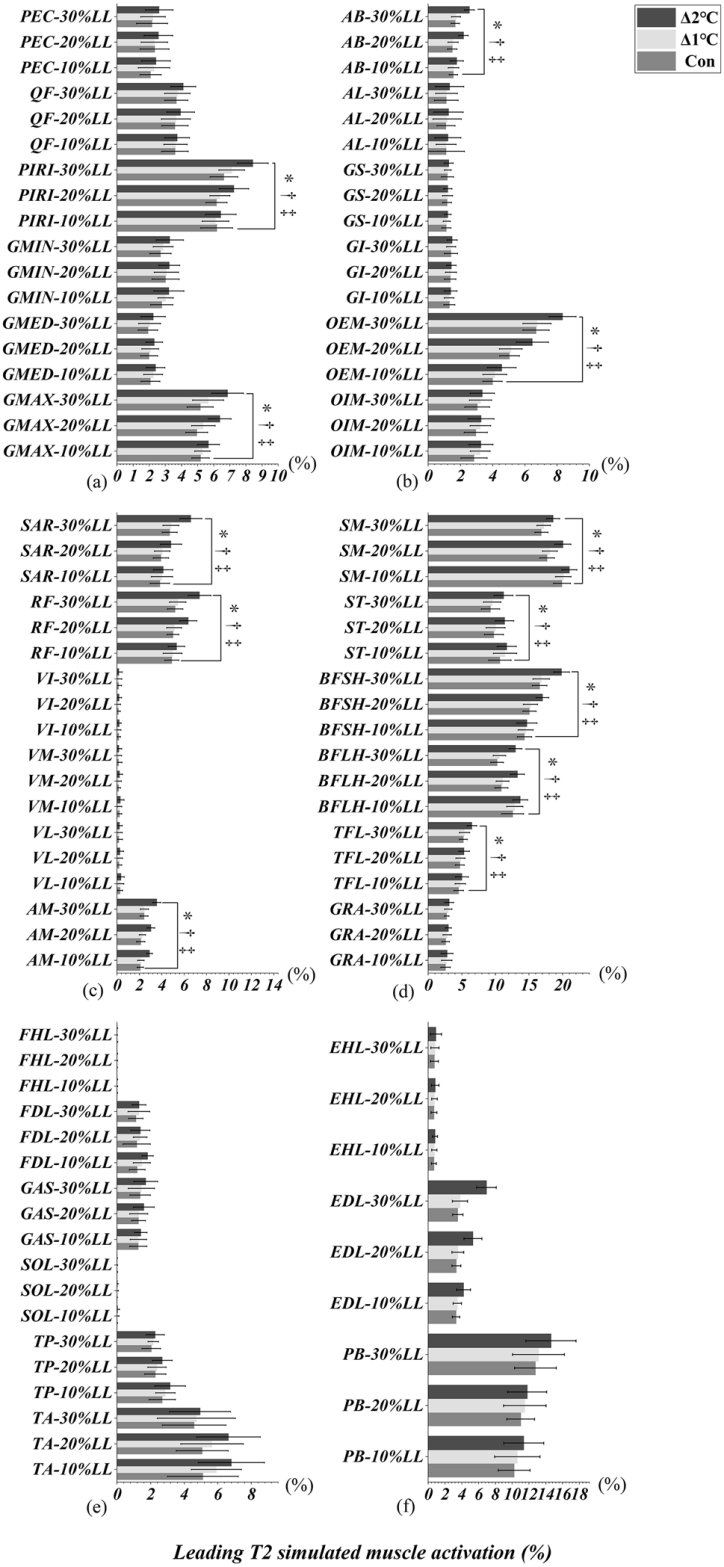
Leading limb simulated muscles activations in T2 (toe-above-obstacle) event with systemic increase of T_oral_ from baseline at obstacle height of 10%, 20% and 30% of leg’s length. “*” Indicates significant T_oral_ x height interaction effects (*p* < 0.05). “†” Indicates a significant difference between Δ2℃ and Con at obstacle height of 20% and 30% leg length (*p* < 0.05). “‡” Indicates a significant difference between Δ2℃ and Δ1℃ at obstacle height of 20% and 30% leg length (*p* < 0.05).

**Figure 5:**
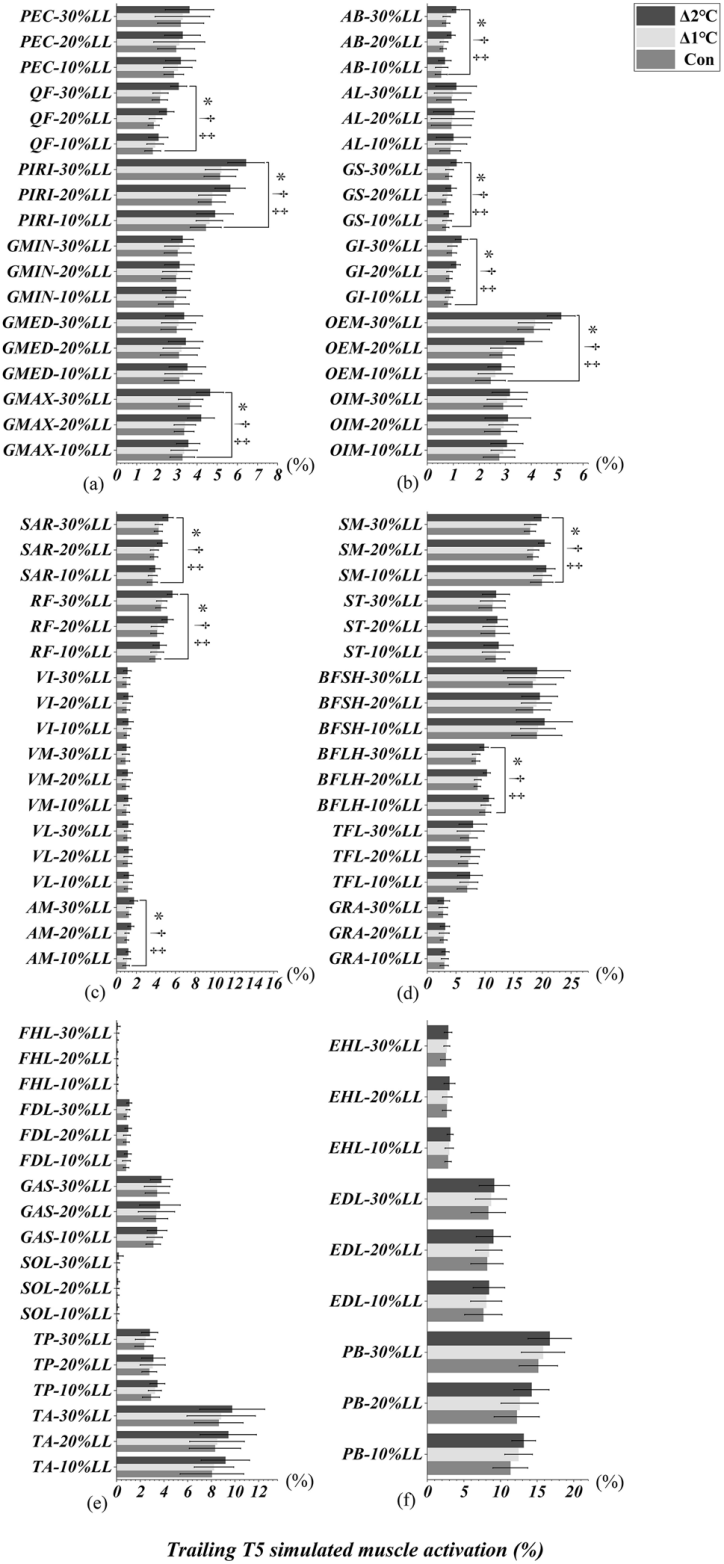
Trailing limb simulated muscles activations in T5 (toe-above-obstacle) event with systemic increase of T_oral_ from baseline at obstacle height of 10%, 20% and 30% of leg’s length. “*” Indicates significant T_oral_ x height interaction effects (*p* < 0.05). “†” Indicates a significant difference between Δ2℃ and Con at obstacle height of 20% and 30% leg length (*p* < 0.05). “‡” Indicates a significant difference between Δ2℃ and Δ1℃ at obstacle height of 20% and 30% leg length (*p* < 0.05).

Figures 4a-d and 5a-d illustrated an interaction effect between T_oral_ and obstacle heights, which were observed in PIRI, GMAX, AB, OEM, SAR, RF, AM, SM and BFLH when leading (Table2) and trailing (Table 2) limbs were in the toe-above-obstacle event (T2/T5, All *p* < 0.046). Specifically, the simple main effect showed that lower limb simulated muscle activation were greater in Δ2°C than in Δ1℃ and Con at obstacle heights of 20% and 30% leg length, respectively (All *p* < 0.001, effect size varying from 0.95 to 3.21, Figs. 4, 5). ST, BFSH, and TFL only interacted when the leading limb was above the obstacle (All *p* < 0.037, Table 2, Fig. 4d). Specifically, the simple main effect showed that the lower limb simulated muscle activation were greater in Δ2°C than in Δ1℃ and Con at an obstacle height of 20% and 30% leg length (All *p* < 0.001, effect size varying from 0.73 to 2.87, Fig. 4d). An interaction effect was also found between T_oral_ and obstacle heights in QF, GI, and GS when the trailing limb was in the toe-above-obstacle event (All *p* < 0.004, Table 2, Fig. 5a, b). Specifically, the simple main effect showed that lower limb simulated muscle activation were greater in Δ2°C than in Δ1℃ and Con at obstacle heights of 20% and 30% leg length (All *p* < 0.001, effect size varying from 0.75 to 2.38, Fig. 5a, b). However, the lower limb simulated muscle activation were not different between Δ1°C and Con (All *p* > 0.05, Figs. 4 and 5).

### Heel-strike event of leading and trailing limbs

**Figure 6:**
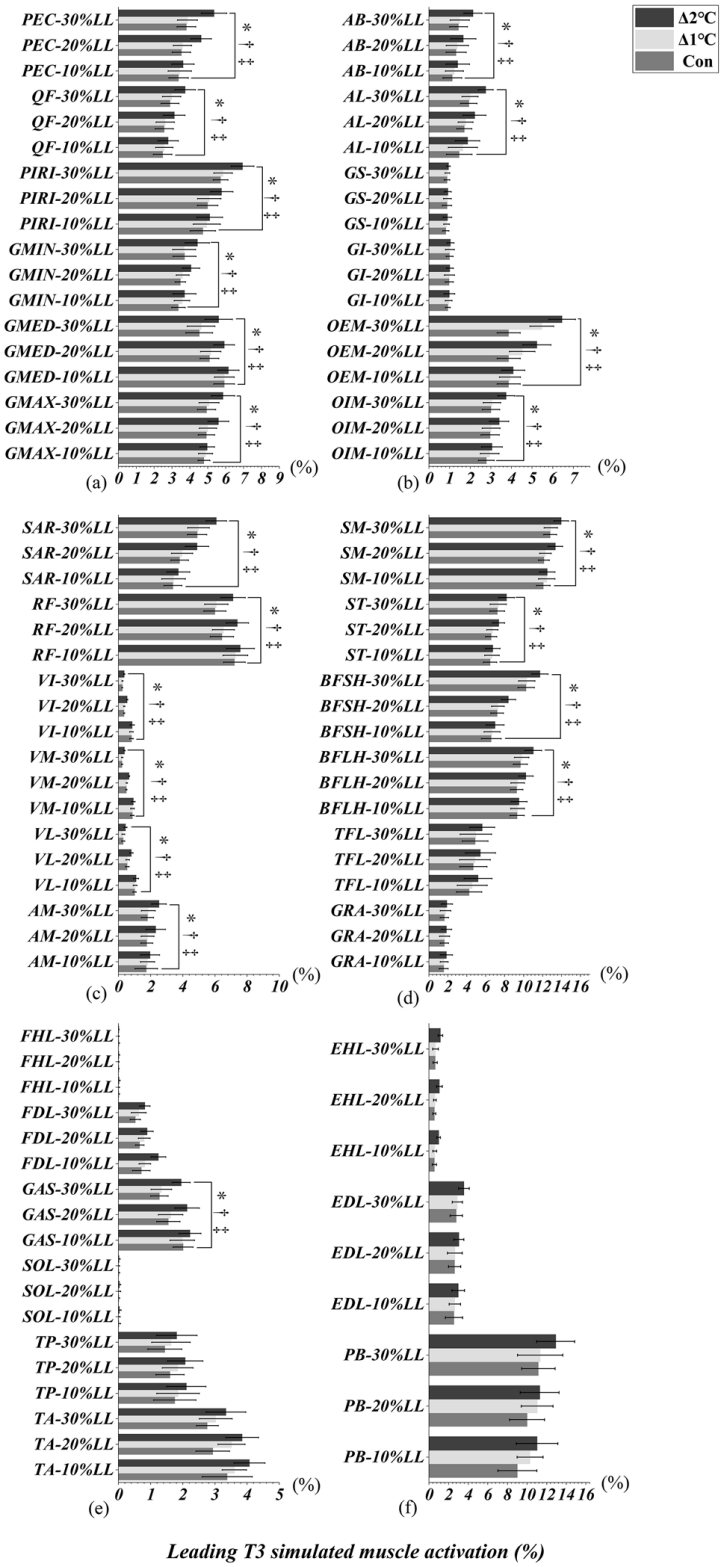
Leading limb simulated muscles activations in T3 (heel-strike) event with systemic increase of T_oral_ from baseline at obstacle height of 10%, 20% and 30% of leg’s length. “*” Indicates significant T_oral_ x height interaction effects (*p* < 0.05). “†” Indicates a significant difference between Δ2℃ and Con at obstacle height of 20% and 30% leg length (*p* < 0.05). “‡” Indicates a significant difference between Δ2℃ and Δ1℃ at obstacle height of 20% and 30% leg length (*p* < 0.05).

**Figure 7:**
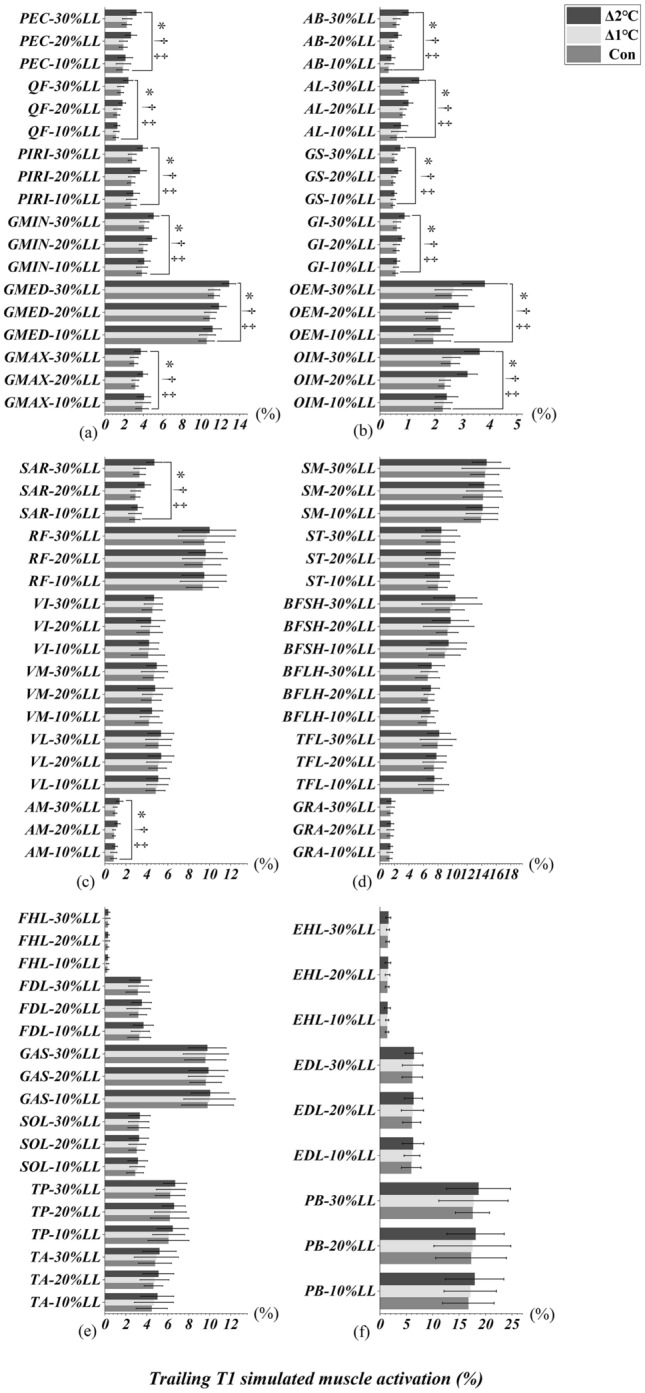
Trailing limb simulated muscles activations in T1 (heel-strike) event with systemic increase of T_oral_ from baseline at obstacle height of 10%, 20% and 30% of leg’s length. “*” Indicates significant T_oral_ x height interaction effects (*p* < 0.05). “†” Indicates a significant difference between Δ2℃ and Con at obstacle height of 20% and 30% leg length (*p* < 0.05). “‡” Indicates a significant difference between Δ2℃ and Δ1℃ at obstacle height of 20% and 30% leg length (*p* < 0.05).

Figures 6a-c and 7a-c illustrated an interactional effect between T_oral_ and obstacle heights were observed in PEC, QF, PIRI, GMIN, GMED, GMAX, AB, AL, OEM, OIM, SAR and AM when the leading (Table 3) and trailing (Table 3) limbs were in the heel-strike event (T3/T1, All *p* < 0.05). RF, VI, VM, VL, SM, ST, BFSH, BFLH, and GAS only interacted when leading limb was in the heel-strike event (All *p* < 0.045, Table 3, Fig. 6c-e). There were also significant interactions in GS and GI when the trailing limb was in the heel-strike event (All *p* < 0.001, Table 3, Fig. 7b). Specifically, the simple main effect showed that simulated muscle activation were greater in Δ2°C than in Δ1℃ and Con at an obstacle height of 20% and 30% leg length when the both leading and trailing limbs were in the heel-strike event (All *p* < 0.001, effect size varying from 0.50 to 4.27). However, lower limb simulated muscle activation were not different between Δ1°C and Con (All *p* > 0.05, Figs. 6 and 7).

## Discussion

This is the first study to examine the rise of T_oral_ on simulated muscle activation of 33 lower limb muscles during crossing obstacles in female participants. After Δ2℃ rise of T_oral_, both leading and trailing limb simulated muscle activation increased when crossing the obstacle height of 20% and 30% leg length to prevent falling during obstacle crossing. Furthermore, Δ1℃ rise in T_oral_ and crossing obstacle height of 10% leg length did not alter lower limb simulated muscle activation. These findings agree with our study hypotheses. Collectively, this study indicates that a greater level of hyperthermia results in a greater lower limb simulated muscle activation at the higher level of the balance task.

### Toe-off event of leading and trailing limbs

In the toe-off event, the activations of pelvic and thigh muscles were greater at Δ2℃ compared to Δ1℃ and Con when the leading and/or trailing limbs crossed the obstacle heights of 20% and 30% leg length. Previous studies showed that QF, PIRI, GI, GS, OEM, and OIM were considered as the “rotator cuff” of the hip, which provided support for the hip joint during gait^24^. The activations of GMIN, GMAX, PEC, and SAR are to ensure the stability of the hip joint and pelvis during walking^25^, so that the trunk and lower limbs are firmly associated with each other during gait. Furthermore, GRA activation can stabilize the external moment to maintain body stability during walk^26^. The greater activations of pelvic and thigh muscles following Δ2℃ of T_oral_ during obstacle crossing at 20% and 30% of obstacle heights could be due to the fact that greater lower limb simulated muscle activation are necessary to compensate the reduction of ankle proprioception due to hyperthermia^5^. Furthermore, ST, BFSH, BFLH are the main actuators in the propulsion phase of walking^27^. After Δ2℃ rise of T_oral_, the leading limb activated ST, BFSH, BFLH to increase the propulsion power of the limbs to improve the success rate of crossing obstacles. GMED stabilizes the pelvis and controls femoral adduction and internal rotation during functional activity, and higher levels of lower limb simulated muscle activation would result in greater stabilization of whole body segments^28^. Adjusting the strength of the adductor muscles during terminal stance can control postural sway of the body^29^ and maintains the stability of the body during gait. Therefore, to further maintain the stability of the body and to reduce the risk of sports-related injuries, the greater activation of GMED, AL, AB during the toe-off event of the trailing limb is deemed necessary.

### Toe-above-obstacle event of leading and trailing limbs

In the toe-above-obstacle event, the activations of pelvic and thigh muscles were greater at Δ2℃ compared to Δ1°C and Con when the leading and/or trailing limbs crossed obstacle with height of 20% and 30% leg length. Previous studies showed that GMAX can be used as a global stabilizer to prevent the trunk from leaning forward^30^. The adductor muscles were involved in controlling the lateral displacement of the pelvis and TFL can act as a pelvic stabilizing muscle^31^. OEM and PIRI reduce the risk of hip dislocation^32^. In this study, leading and trailing limbs increased PIRI, GMAX, AB, OEM, SAR, RF, AM, SM, and BFLH activations to stabilize the crossing limbs and trunk to ensure smooth crossing of the obstacle in toe-above-obstacle event in the Δ2°C compared to Δ1°C and Con. Furthermore, knee flexion is particularly important to increase toe-clearance^33^, the increase of toe clearance can reduce falling risk^34^. ST, SM, BFLH, BFSH, and SAR are the major agonists to flex the knee joint and thus to ensure safety crossing of the obstacle without falling^35^. Furthermore, RF was active during the swing phase of walking to prevent excessive knee flexion stable^36^. After 2℃ rises of T_oral_, the activations of ST, SM, BFLH, BFSH, SAR, and RF were greater to increase Toe-clearance and maintain the stability of the knee joint to reduce falling risk. Moreover, QF, GI, and GS are external rotators of the short hip, which provide external rotation torque and mechanical stability for the hip joint^37^ to enhance the hip joint stability during crossing obstacles.

### Heel-strike event of leading and trailing limbs

In the heel-strike event, the activations of pelvic, thigh and posterior calf muscles were greater at Δ2℃ compared to Δ1℃ and Con when the leading and trailing limbs crossed obstacle with height of 20% and 30% leg length. Previous studies showed that the activation of hip muscles during heel strike event can increase lower limb coordination^38^. In this study, the activations of the leading and trailing hip muscles were greater to enhance the simulated muscle activation of lower limb to increase the control of the lower limb after 2℃ rises of T_oral_ (Figs. 6, 7). In addition, hip adductors in the first half of stance accelerate the body and maintain hip motion and stability^39^. QF, PIRI,OEM, OIM, GS, and GI are the external rotators of the hip joints, when combined with their rotational antagonists (GMIN, PEC, SAR) to provide hip joint stability^24^. After Δ2℃ rises of T_oral_, greater activation of the hip adductors and external rotators may promote joint stability to prepare for the conversion of the supporting limb during heel-strike events. Moreover, previous studies indicated that quadriceps (RF, VI, VM, VL) and hamstrings (SM, ST, BFSH, BFLH) slow the forward propulsion and provide vertical support during the early stance phase, and there is a compensatory mechanism between quadriceps and hamstrings at the end of swing phase to prepare the knee for landing^40,41^. The gastrocnemius and quadriceps can stabilize the knee joint during weight-bearing activity^42^. Therefore, greater activation of quadriceps, hamstrings, and GAS during the heel strike event greatly reduced the impact loading of the knee joint and increased limb stability to reduce postural sway after Δ2℃ rise of T_oral_.

While we have successfully addressed the systematic rise of T_oral_ on lower extremity muscles activation during obstacle crossing at various heights in female participants, this study has three major limitations. First, unnecessary muscle co-contraction caused by muscle redundancy may exist in the neuromuscular system, resulting in multiple muscle coordination patterns that may affect the results of muscle simulations. Secondly, this study used a whole-body musculoskeletal model, but so far only the degree of simulated muscle activation of the lower extremities has been explored, the effect of crossing obstacles after T_oral_ rise on whole-body muscles simulation has not been analyzed. Thirdly, we did not address the the effect of menstrual cycle with different rise of T_oral_ on lower extremity simulated muscle activation during obstacle crossing at various heights. This issue is considered important especially given the fact that the resting T_oral_ was 0.3–0.5℃ higher at the luteal phase compared to the early follicular phase, which could potentially result in a higher lower extremity simulated muscle activation during obstacle crossing at Δ2°C. This issue therefore warrants further investigation. However, this issue is not directly related to the main purpose of this study, and we are also confident that the effect of the menstrual cycle would only affect simulated muscle activation when T_oral_ rise is greater or equal than 2°C as we observed lower extremity simulated muscle activation was not different between 1°C and 2°C as well as between 1°C and Con. Lastly, we acknowledged that we did not measure core temperature using rectal or esophageal which could be potentially more accurate in terms of quantifying body temperature. However, since oral temperature has been previously used in passive heating research^43–45^, we believe this would not affect the primary outcome of this study like muscle simulation.

## Conclusion

We showed that when T_oral_ increased by Δ2℃, the simulated muscle activation of both leading and trailing limbs were greater in the toe-off, toe-above-obstacle, and heel-strike events when crossing an obstacle with height of 20% or 30% leg length. Therefore, when increase T_oral_ by 2°C led to greater balance instability and increased simulated muscle activation in the lower limbs compared to Δ 1°C and CON, facilitating safely obstacles crossing.

### Supplementary Information

Supplementary Information 1.

Supplementary Information 2.

## Data Availability

The datasets generated during and/or analysed during the current study are available from the corresponding author on reasonable request.
